# Supplementary figures and images for: Molecular basis of F-actin regulation and sarcomere assembly via myotilin
Source: PLoS Biol. 2021 Apr 12;19(4):e3001148. doi: 10.1371/journal.pbio.3001148 (PMC8062120; doi:10.1371/journal.pbio.3001148)

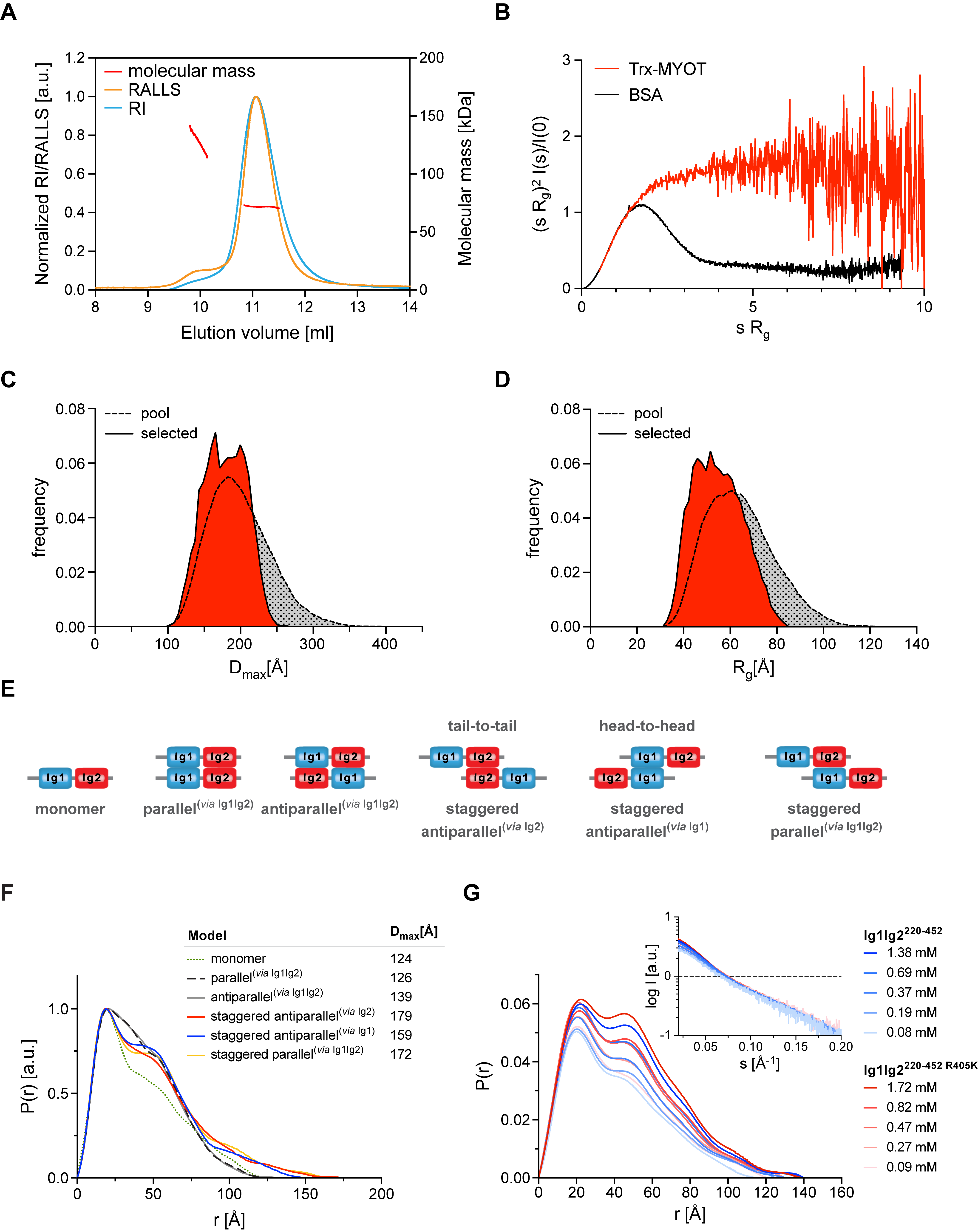

Supplement: S1 Fig — Myotilin displays conformational ensemble in solution. (A) Determination of molecular mass across the RI/RALLS elution peak collected in parallel to SEC-SAXS mode. Molecular mass estimates are shown in red, RALLS trace in orange and RI trace in blue. Molecular mass across the main elution peak is 70 ± 7 kDa. Molecular mass estimates corresponding to elution time points prior to the main elution peak suggest a small presence of higher oligomeric species such as dimers. (B) Dimensionless Kratky plot indicates that while Trx-MYOT does contain ordered regions (corresponding to the Trx moiety and the Ig1 and Ig2 domains), most of the polypeptide chain is disordered and extended. In comparison, dimensionless Kratky plot of BSA, representing a typical folded protein, is shown. (C and D) Dmax and Rg distributions from EOM analysis of Trx-MYOT. The parameters derived from the selected pool compared to the original pool suggest that Trx-MYOT is slightly restrictive in its flexibility and occupies a more compact conformation (EOM fit and models are shown in Fig 1C and 1E). (E) Schematic representation of Ig1Ig2220–452 depicting potential modes of dimerization. (F) Theoretical P(r) vs. r plots calculated for potential Ig1Ig2220–452 dimerization modes showed in (E). Of these, both parallel and antiparallel dimers using tandem Ig1Ig2 as dimerization interface (parallel(via Ig1Ig2), antiparallel(via Ig1Ig2)) display marginal increase in the Dmax. Staggered dimers display notable increase in the Dmax similar to experimentally observed (Fig 1F, S1 Table). In comparison to experimental data, where Ig1Ig2220–452 adopts a conformational ensemble in solution (Fig 1E) [23], P(r) vs. r plots for different dimerization modes were calculated using 1 (static) conformation, consequently resulting in a longer Dmax compared to experimentally derived. In order to compare various P(r) functions, P(r) was normalized to the peak height. (G) P(r) vs. r plot for the concentration series of Ig1Ig2220– [file pbio.3001148.s001.tif]

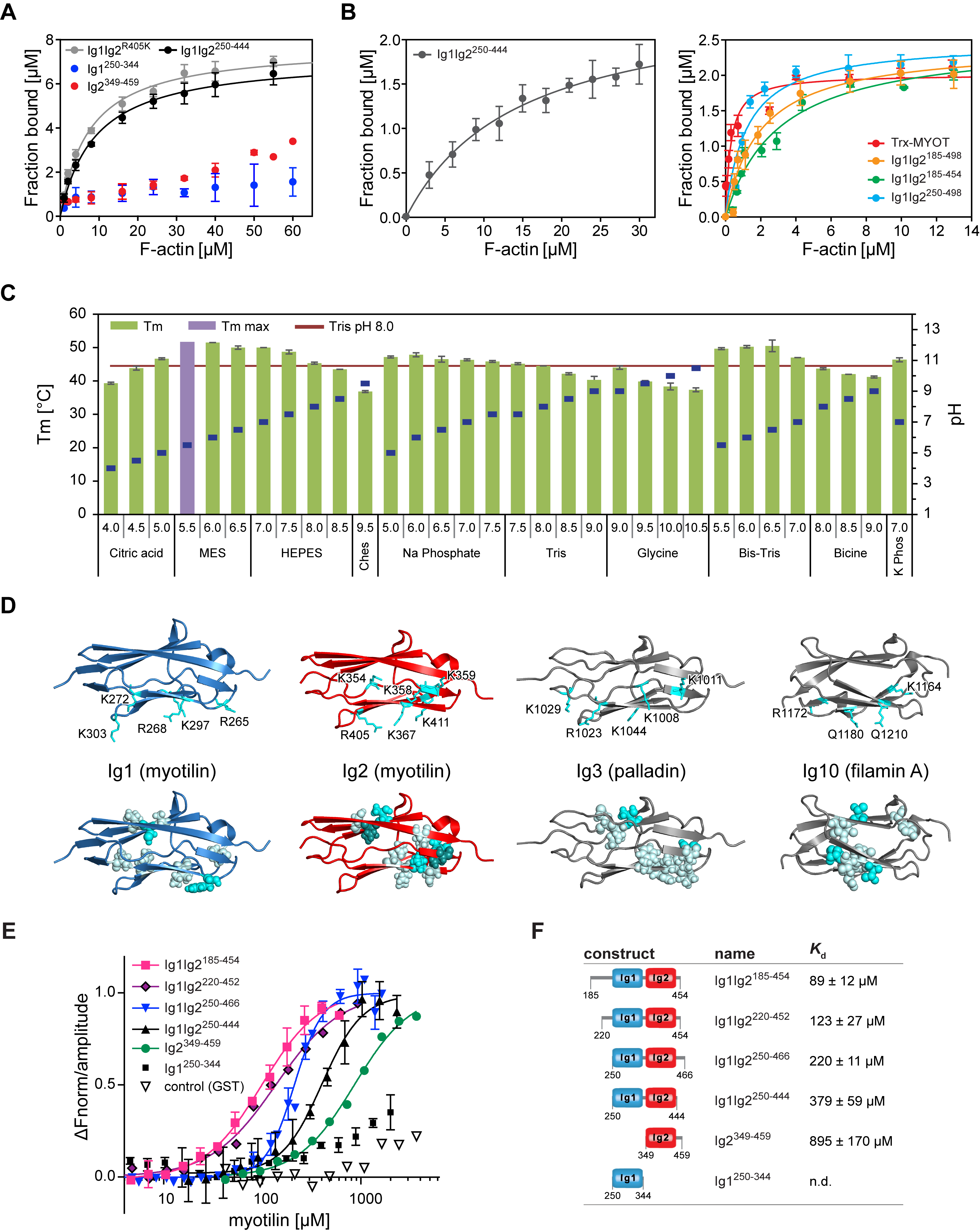

Supplement: S2 Fig — Myotilin binds to F-actin via Ig domains and disordered flanking regions and influences its dynamics. (A and B) Binding of various myotilin constructs to F-actin in conditions B1 (A) or conditions B2 (B). The exponential binding curves fitted for each set of data points were used to determine apparent binding affinities shown in Fig 2A. Plotted values represent the mean ± SEM from 3 to 5 independent experiments. (C) Melting temperature (Tm) of Trx-MYOT in various buffers as assessed by DSF assay (for details, see Materials and methods). The pH of each buffer (blue rectangle) is indicated. Tm max (violet) denotes the buffer conditions in which Tm of Trx-MYOT was the highest (100 mM MES, pH 5.5). Brown line indicates Tm in the standard buffer (100 mM Tris, pH 8.0). Note trend in increasing the stability of Trx-MYOT by lowering the pH. (D) Structural analysis of the functional (F-actin binding) sites on various Ig domains. Top panel: Basic amino acid residues with surface-exposed sidechains are shown as sticks (cyan). For palladin and filamin A, this region corresponds to the F-actin binding region. Bottom panel: The functionally important residues as predicted by evolutionary coupling analysis and folding server EVfold [30] are shown as spheres and coincide with the residues shown in the top panel on the lateral sides of the Ig domains. More intense (darker) color depicts a higher probability that the residue represents a functional site. (E) Binding of various myotilin constructs and control (GST) to fluorescently labeled monomeric DVD-actin measured by MST. (F) Table of the constructs and their affinity to DVD-actin obtained from the data shown in (E). Data points that were used to create graphs are reported in S2 Data. DSF, differential scanning fluorimetry; MST, microscale thermophoresis; Trx, thioredoxin. (TIF) [file pbio.3001148.s002.tif]

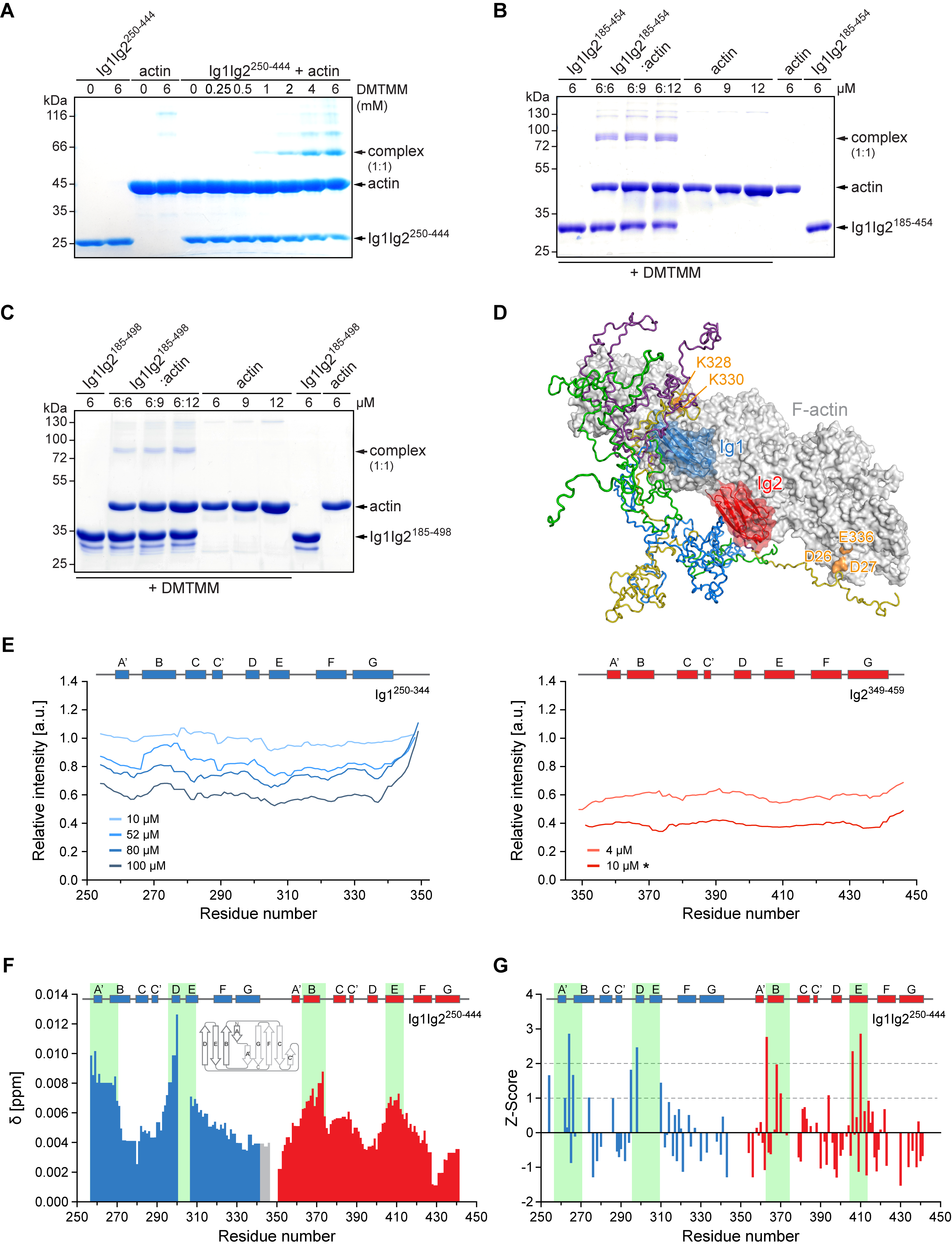

Supplement: S3 Fig — Integrative model of myotilin:F-actin complex. (A–C) Cross-linking of myotilin Ig1Ig2250–444 (A), Ig1Ig2185–454 (B), and Ig1Ig2185–498 (C) with F-actin. Myotilin-actin complexes were cross-linked with DMTMM, either at increasing concentrations of DMTMM and fixed amount of proteins (Ig1Ig2250–444) or at its fixed concentration and varying amounts of proteins (Ig1Ig2185–454 and Ig1Ig2250–498). SDS-PAGEs of cross-linked samples stained with Coomassie Brilliant Blue are shown. Specific bands, corresponding to 1:1 complex, were analyzed by MS, and data obtained from it are summarized in S2 Table. The uncropped gels can be found in S1 Data. (D) Model of the full-length myotilin bound to F-actin. Ig domains of myotilin were docked on F-actin and combined with flexible (not docked) models of N-terminal and carboxyl-terminal parts of the protein from EOM analysis of Trx-MYOT (Fig 1E). The 4 most populated conformations of N-terminal and carboxyl-terminal parts are shown in distinct colors. Selected residues of actin found in cross-links with myotilin are shown in orange. See also Fig 3 and S2 Table. (E) Relative change of 1H-15N HSQC cross-peak intensities upon addition of F-actin to Ig1250–344 (blue, left panel) or Ig2349–459 (red, right panel). Values were averaged by a sliding window function over 11 amino acids. Stronger reduction in signal intensity of Ig2349–459 as compared to Ig1250–344 indicates that Ig2 binds more tightly to F-actin than Ig1. In all experiments, except for the one indicated by an asterisk (67 μM), a fixed concentration of myotilin was used (100 μM), while the concentration of F-actin was varied as indicated in the figure. (F) 1H-15N HSQC monitored shift changes in Ig1Ig2250–444 (50 μM) upon addition of F-actin (4 μM). Shifts were averaged by a sliding window function over 11 amino acids. In Ig1Ig2250–444, 4 main regions with the most pronounced effect upon addition of F-actin were identified (boxed green). Inset, topology diagram of I-type Ig domain [file pbio.3001148.s003.tif]

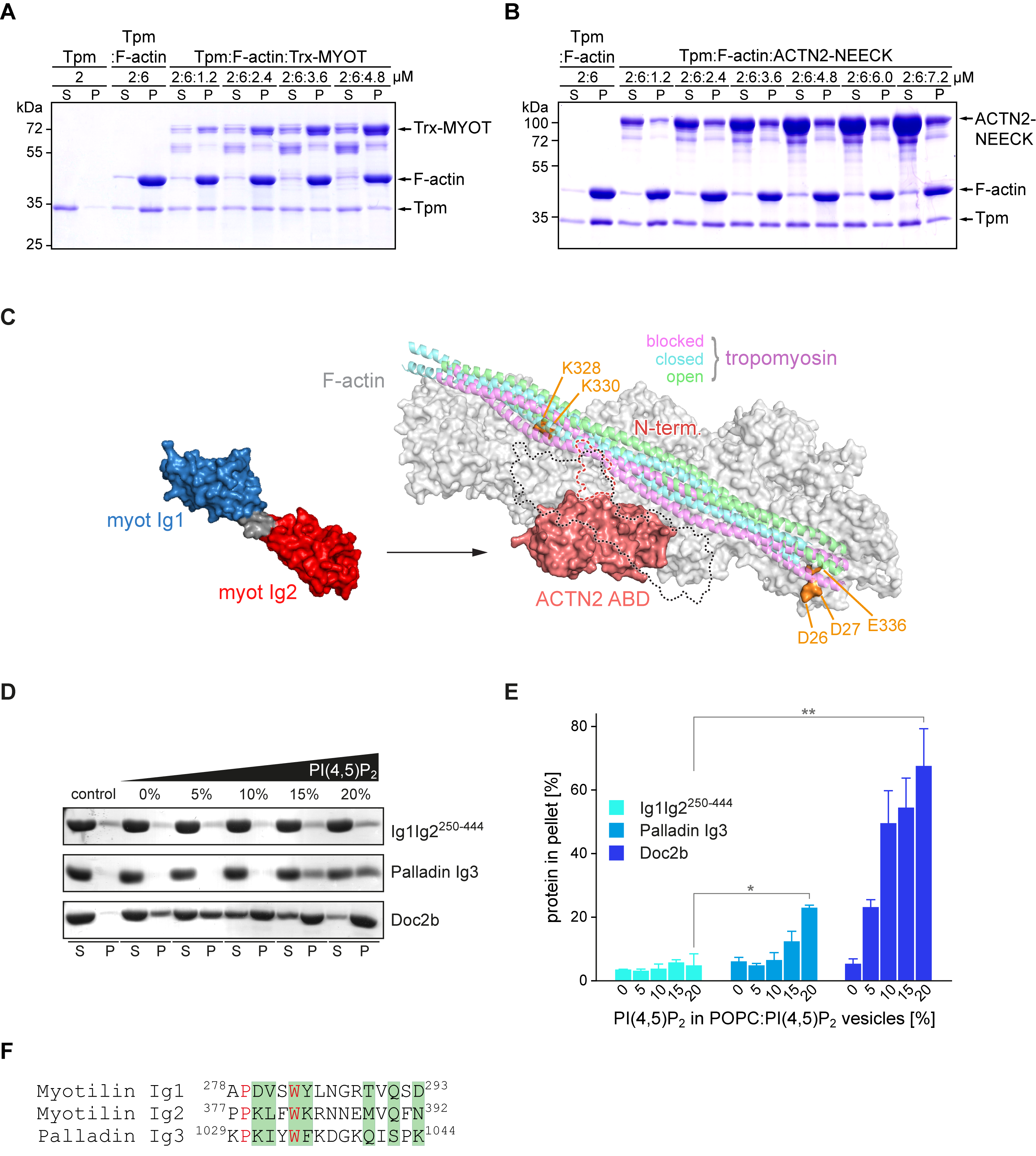

Supplement: S4 Fig — Myotilin regulates binding of tropomyosin to F-actin and does not interact with PI(4,5)P2. (A) Effects of myotilin on tropomyosin:F-actin interaction. Example of the SDS-PAGE used to generate Fig 4B is shown. Tpm was incubated with F-actin before addition of Trx-MYOT at molar ratios indicated on the figure. F-actin and proteins bound were sedimented by centrifugation, and equal amounts of supernatant (S) and pellet (P) fractions were subjected to SDS-PAGE. (B) Effect of myotilin on α-actinin:F-actin interaction. Example of the SDS-PAGE used to generate Fig 4D is shown. ACTN2-NEECK was incubated with F-actin before addition of Tpm at molar ratios indicated on the figure. F-actin and proteins bound were sedimented by centrifugation, and equal amounts of supernatant (S) and pellet (P) fractions were subjected to SDS-PAGE. (C) Model of tandem Ig domains of myotilin, ACTN2 ABD, and tropomyosin bound to F-actin was generated by superposition of our myotilin:tropomyosin:F-actin model shown on Fig 4A with the structures of F-actin bound to α-actinin-2, spectrin and filamin A, and ABDs, at nominal resolutions of 16, 6.9, and 3.6 Å, respectively [38–40]. In the next step, crystal structure of α-actinin-2 ABD (salmon color, PDB: 5A36) [85] was superimposed over ABD of filamin A (homologous to ABD of cardiac and skeletal muscles expressed isoform filamin C) to obtain the final model. Dotted line (black) indicates position of tandem Ig domains of myotilin (myot Ig1, myot Ig2) on F-actin as shown on Fig 4A. N-terminal regions preceding the ABDs increase affinity to F-actin and are isoform specific [39]. The potential trace of the α-actinin-2 N-terminal extension (N-term.) on F-actin, as deduced from high-resolution structures of F-actin decorated by filamin A and spectrin ABDs is shown with a dashed red line [40]. In addition, actin-binding sites of α-actinin-2 and myotilin clearly overlap, explaining mutually exclusive binding of myotilin, α-actinin-2 and tropomyosin to F-actin. [file pbio.3001148.s004.tif]

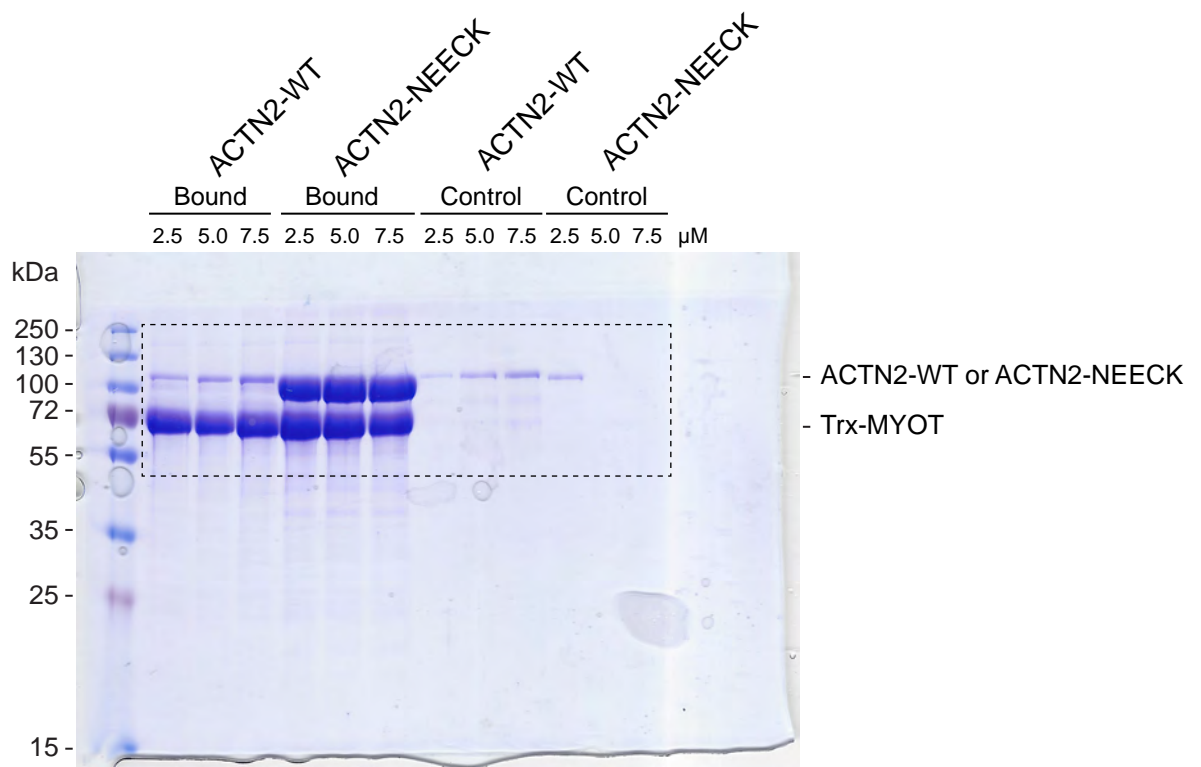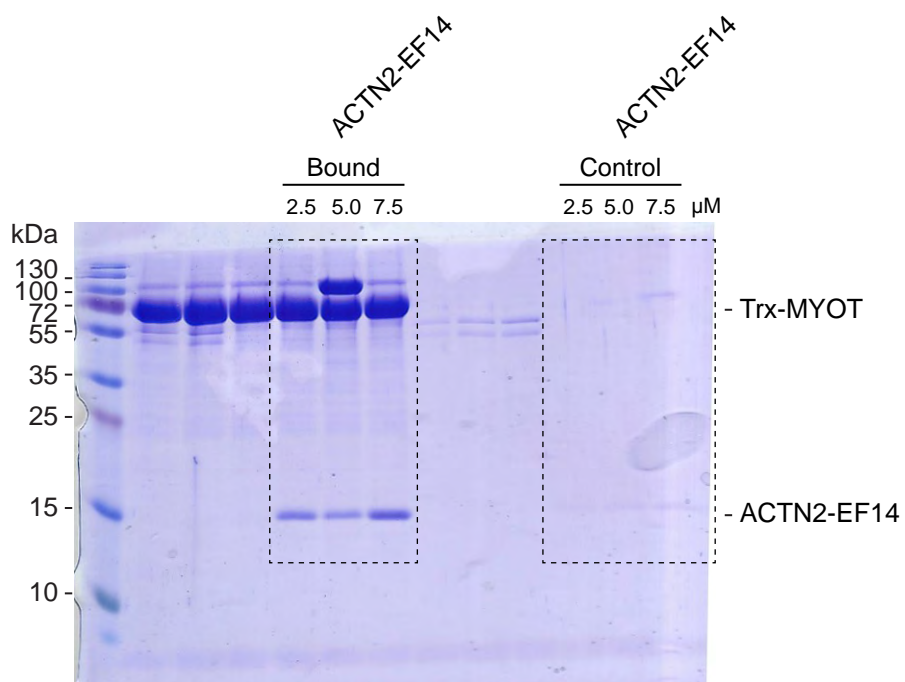

Fig 4F

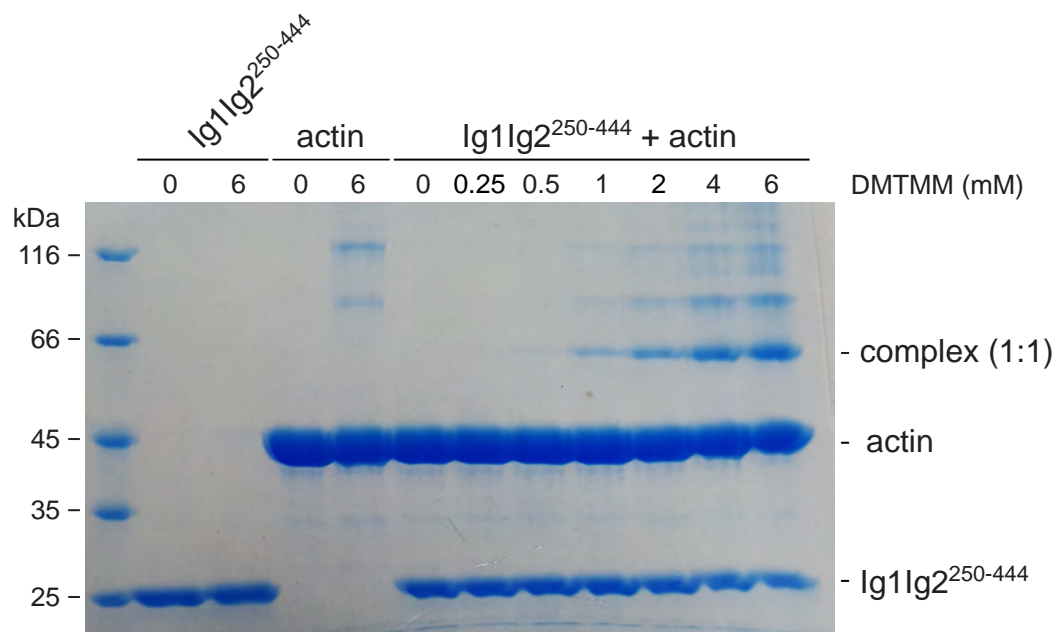

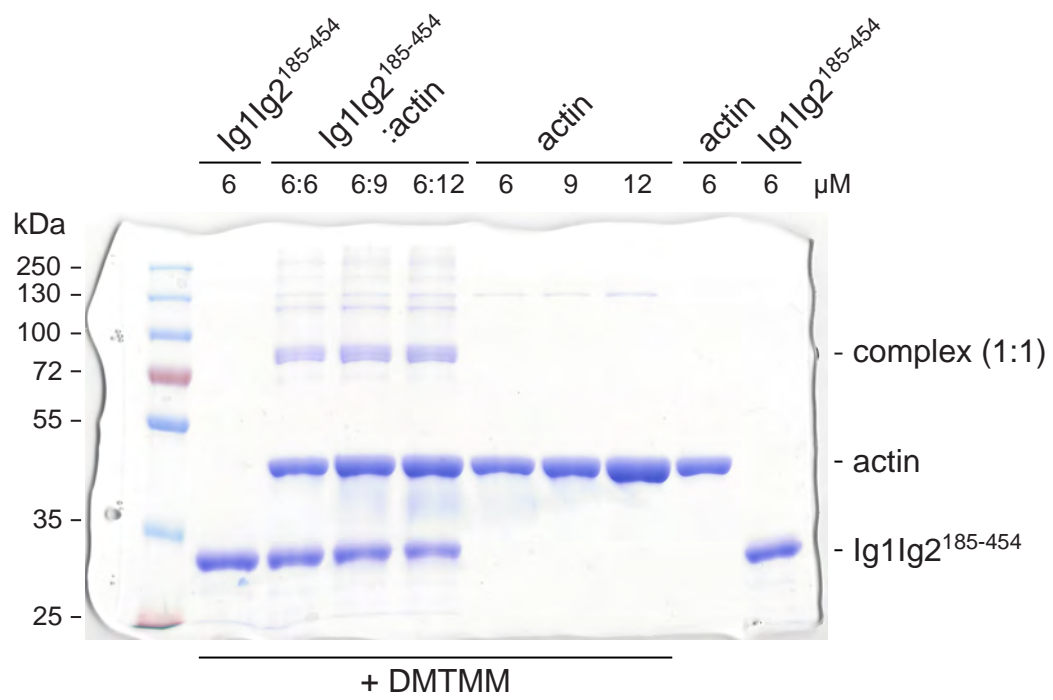

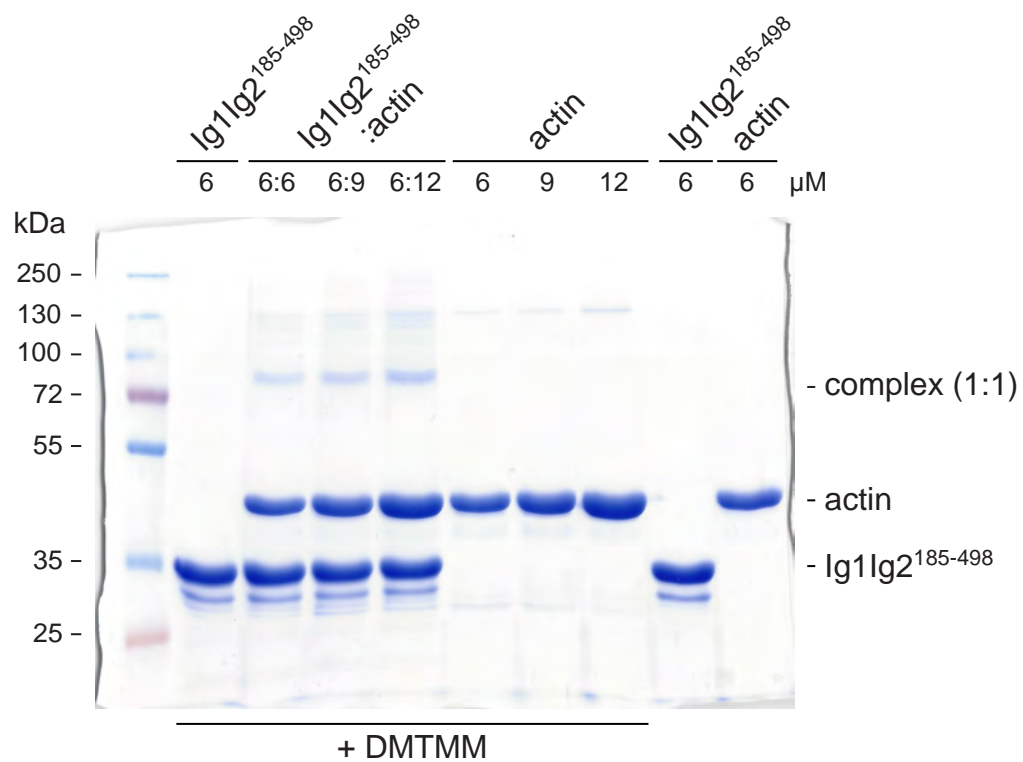



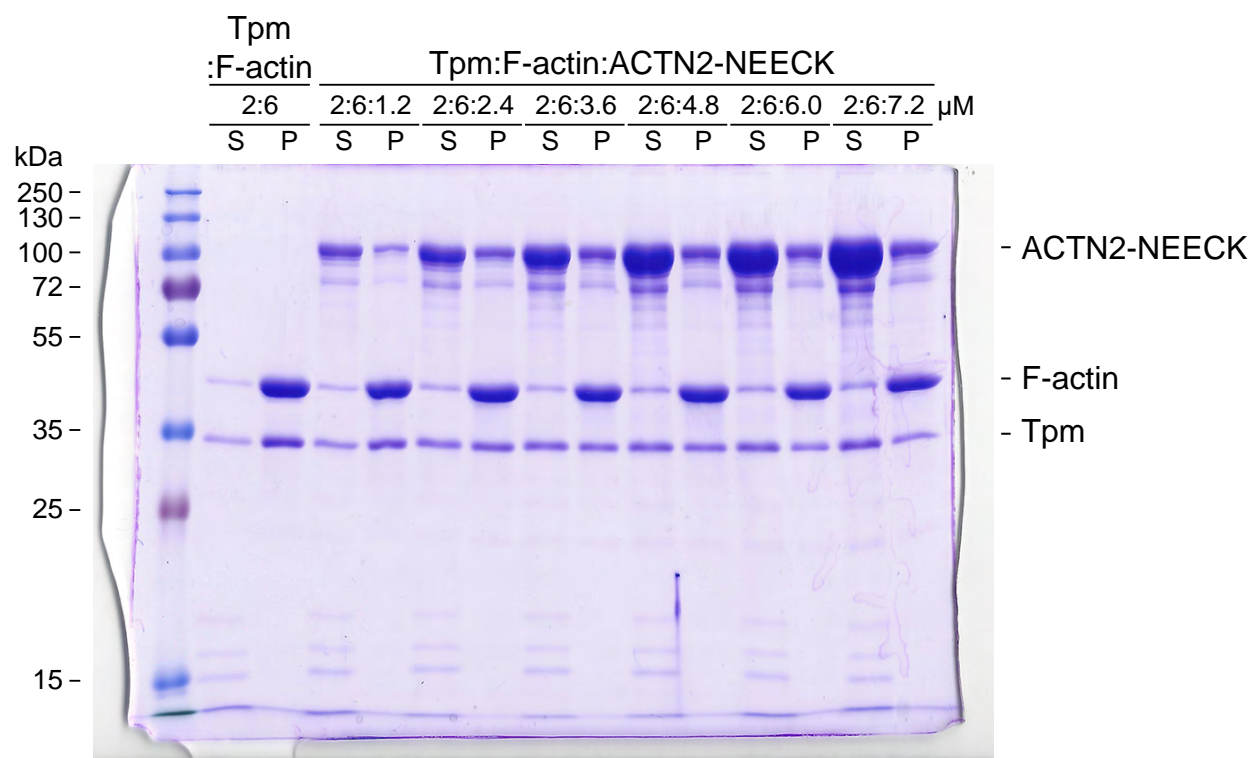

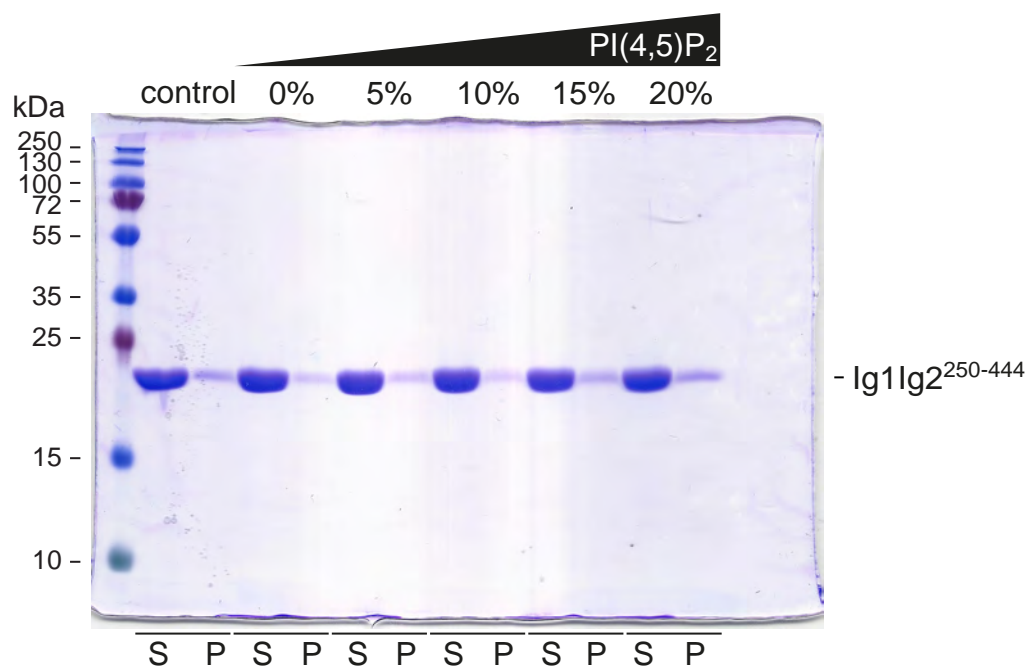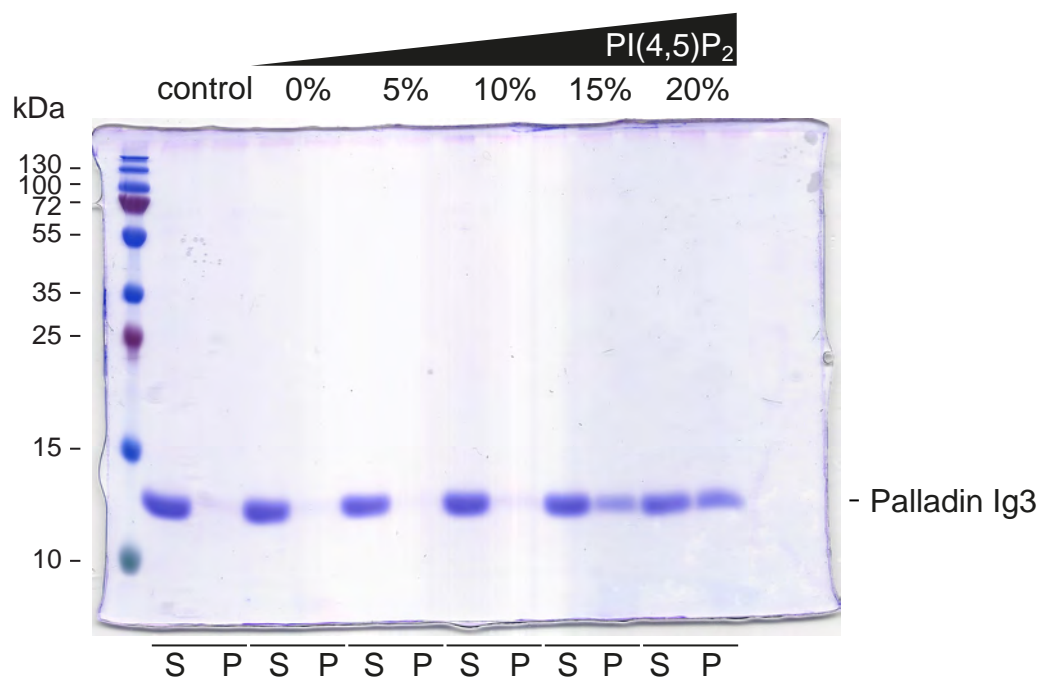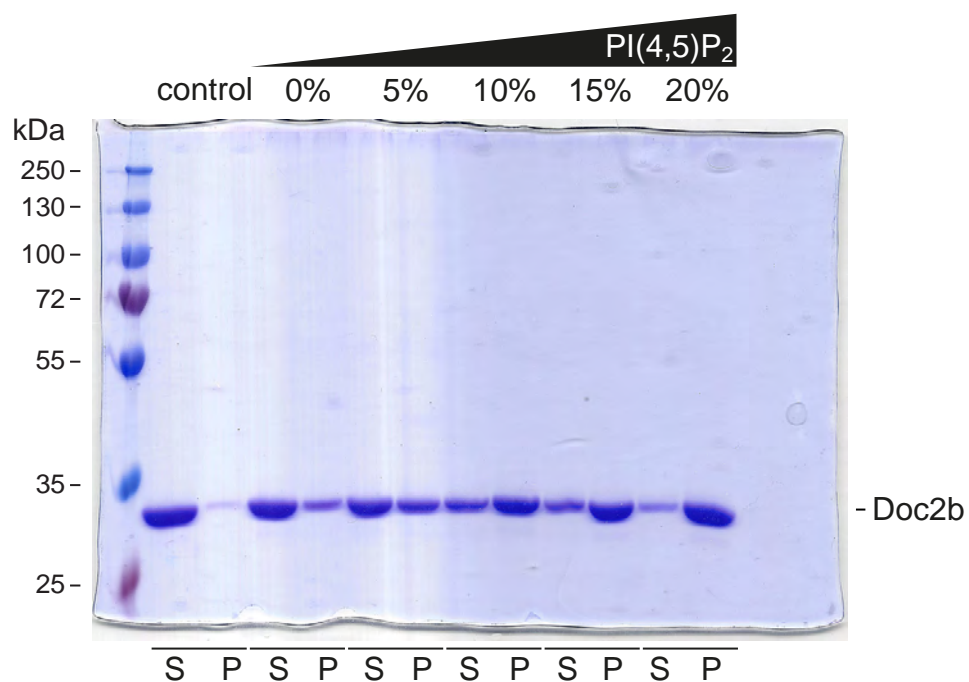

Supplement: S1 Data — Uncropped gels used to generate Fig 4F and S3A–S3C, S4A, S4B, and S4D Figs. (PDF) [file pbio.3001148.s005.pdf]
