# Supplementary material for: Molecular basis of F-actin regulation and sarcomere assembly via myotilin
Source: PLoS Biol. 2021 Apr 12;19(4):e3001148. doi: 10.1371/journal.pbio.3001148 (PMC8062120; doi:10.1371/journal.pbio.3001148)
Supplement: S1 Table — (PDF) [file pbio.3001148.s007.pdf]

S1 Table. SAXS data collection, analysis and derived structural parameters

Construct	Ig1Ig2$^{250\text{-}444}$*	Ig1Ig2$^{250\text{-}498}$	Ig1Ig2$^{220\text{-}452}$	Trx-MYOT	Ig1Ig2$^{220\text{-}452\dagger}$	Ig1Ig2$^{220\text{-}452\ R405K}$
**Data collection parameters**						
Radiation source	ESRF (Grenoble, France)		DESY (Hamburg, Germany)			
			Doris	Petra III		
Beamline	BM29 BioSAXS		EMBL X33	EMBL P12		
Detector	Pilatus 1M		Pilatus 1M-W	Pilatus 2M	Pilatus 6M	
Beam geometry [mm, FWHM]	0.10 × 0.20		n.d.	0.12 × 0.20	0.12 × 0.20	
Wavelength [nm]	0.099		0.126	0.124	0.124	
Sample–detector distance [m]	2.867		2.7	3.1	3.0	
Momentum of transfer *s* range [nm^{-1}]	0.04–4.0		0.1–6.0	0.08–3.5	0.15–7.3	
Exposure time [s]	15		15	1 (SEC-SAXS)	0.145	
Temperature [°C]	20		11	20	20	
Buffer	20 mM HEPES, 150 mM NaCl, 5% glycerol, 1 mM DTT, pH 7.4		20 mM MES, 200 mM NaCl, 3% glycerol, pH 6.0	20 mM Tris 400 mM NaCl, 250 mM arginine, 5% glycerol, pH 7.5	20 mM HEPES, 150 mM NaCl, 5% glycerol, 1 mM DTT, pH 7.4	
**Overall parameters**						
Conc. range measured$^{\$}$	1–14.12 mg/ml (0.05–0.64 mM)	1–42.8 mg/ml (0.04–1.53 mM)	1–52 mg/ml (0.04–1.96 mM)	n.a.	2.1–36.6 mg/ml (0.08–1.38 mM)	2.3–45.5 mg/ml (0.09–1.72 mM)
R_g (Guinier) [Å]	28 (0.64 mM)	32 (0.54 mM)<br>37 (1.53 mM)	31 (0.04 mM)<br>37 (0.79 mM)<br>39 (1.96 mM)	51	30 (0.08 mM)<br>33 (0.19 mM)<br>33 (0.37 mM)<br>34 (0.69 mM)<br>36 (1.38 mM)	33 (0.09 mM)<br>34 (0.27 mM)<br>34 (0.47 mM)<br>35 (0.82 mM)<br>36 (1.72 mM)
R_g from PDDF [Å]	29 (0.64 mM)	34 (0.54 mM)<br>38 (1.53 mM)	32 (0.04 mM)<br>39 (0.79 mM)<br>41 (1.96 mM)	53	33 (0.08 mM)<br>34 (0.19 mM)<br>35 (0.37 mM)<br>37 (0.69 mM)<br>37 (1.38 mM)	34 (0.09 mM)<br>36 (0.27 mM)<br>36 (0.47 mM)<br>37 (0.82 mM)<br>38 (1.72 mM)
D_{max} [Å]	101 (0.64 mM)	140 (0.54 mM)<br>152 (1.53 mM)	107 (0.04 mM)<br>151 (0.79 mM)<br>152 (1.96 mM)	200	115 (0.08 mM)<br>125 (0.19 mM)<br>130 (0.37 mM)<br>140 (0.69 mM)<br>140 (1.38 mM)	120 (0.09 mM)<br>130 (0.27 mM)<br>140 (0.47 mM)<br>140 (0.82 mM)<br>140 (1.72 mM)
Molecular mass (RALLS) [kDa]	n.a.	n.a.	n.a.	70	n.a.	n.a.
Molecular mass (DATMOV) [kDa]	24.3 (0.64 mM)	31.2 (0.54 mM)<br>44.7 (1.53 mM)	27.3 (0.04 mM)<br>44.7 (0.79 mM)<br>58.1 (1.96 mM)	63.7	30.8 (0.08 mM)<br>30.7 (0.19 mM)<br>33.7 (0.37 mM)<br>37.8 (0.69 mM)<br>43.2 (1.38 mM)	29.1 (0.09 mM)<br>31.9 (0.27 mM)<br>35.6 (0.47 mM)<br>40.6 (0.82 mM)<br>45.7 (1.72 mM)

(continued)

(continued)

Construct	Ig1Ig2$^{250-444*}$	Ig1Ig2$^{250-498}$	Ig1Ig2$^{220-452}$	Trx-MYOT	Ig1Ig2$^{220-452\dagger}$	Ig1Ig2$^{220-452\ R405K}$
Molecular mass from Porod volume [kDa]	21.4 (0.64 mM)	26.4 (0.54 mM)<br>37.9 (1.53 mM)	27.5 (0.04 mM)<br>36.7 (0.79 mM)<br>48.2 (1.96 mM)	77.3	26.9 (0.08 mM)<br>25.9 (0.19 mM)<br>28.3 (0.37 mM)<br>30.9 (0.69 mM)<br>36.2 (1.38 mM)	24.5 (0.09 mM)<br>26.3 (0.27 mM)<br>29.6 (0.47 mM)<br>33.6 (0.82 mM)<br>38.2 (1.72 mM)
Molecular mass (monomer sequence) [kDa]$^{\&}$	21.9	28.0	26.5	69.7	26.5	26.5
**Software employed**						
Primary data red.	SaxsAnalysis pipeline system			SASFLOW	SaxsAnalysis pipeline system	
Data processing	PRIMUS			PRIMUS / Cromixs	PRIMUS	
Calculation and comparison of scattering data	Crysol / Oligomer				n.a.	
Ab initio modelling	DAMMIF				n.a.	
Addition of missing residues	CORAL				n.a.	
Ensemble modelling	EOM				n.a.	
SASDB accession code	SASDF38	SASDF48	SASDF28	SASDFZ7	SASDJH8	SASDJJ8

*, the data for Ig1Ig2$^{250-444}$ were already measured and described previously [23]

$, for Ig1Ig2$^{250-498}$ and Ig1Ig2$^{220-452}$ structural parameters at various concentrations, relevant for the comparison, were calculated separately

&, Molecular mass (monomer sequence) denotes molecular mass calculated from the amino acid sequence of the monomeric species

†, measured at the same experimental settings/conditions as Ig1Ig2$^{220-452\ R405K}$
