# Supplementary material for: Molecular basis of F-actin regulation and sarcomere assembly via myotilin
Source: PLoS Biol. 2021 Apr 12;19(4):e3001148. doi: 10.1371/journal.pbio.3001148 (PMC8062120; doi:10.1371/journal.pbio.3001148)
Supplement: S2 Table — (PDF) [file pbio.3001148.s008.pdf]

S2 Table. List of major cross-links found between myotilin and F-actin

Myotilin constructs used **(A)** 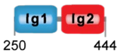

(B) 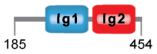

(C) 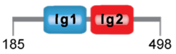

From Protein	From Residue	To Protein	To Residue	Myotilin region	Myotilin fragment identified	Score
myotilin	**K 474**	actin	**E 102**	C-ter.	C	212.6
myotilin	**K 462**	actin	**D 27**	C-ter.	C	199.5
myotilin	**K 411**	actin	**D 27***	Ig2	A, B	A) 157.5, B) 175.5
myotilin	**K 469**	actin	**E 102**	C-ter.	C	169.4
myotilin	**D 236**	actin	**K 330**	N-ter.	B, C	B) 138.4, C) 76.2
myotilin	**D 236**	actin	**K 328***	N-ter.	B	134.9
myotilin	**K 246**	actin	**E 336**	N-ter.	B, C	B) 105.9, C) 111.2
myotilin	**E 245**	actin	**K 330**	N-ter.	B	107.1
myotilin	**K 246**	actin	**D 26**	N-ter.	C	106.3
myotilin	**K 452**	actin	**D 27***	C-ter.	C	103.9
myotilin	**D 241**	actin	**K 330***	N-ter.	B	102.4
myotilin	**K 469**	actin	**D 26***	C-ter.	C	102.0
myotilin	**K 452**	actin	**D 26**	C-ter.	C	100.9
myotilin	**D 239**	actin	**K 330***	N-ter.	B	97.0
myotilin	**D 239**	actin	**K 328**	N-ter.	B	88.1
myotilin	**K 474**	actin	**D 27**	C-ter.	C	84.6
myotilin	**K 474**	actin	**E 336***	C-ter.	C	82.7
myotilin	**D 241**	actin	**K 328***	N-ter.	B	78.2
myotilin	**K 462**	actin	**D 26***	C-ter.	C	77.7
myotilin	**K 354**	actin	**D 27***	Ig2	C	76.7
myotilin	**K 246**	actin	**D 27***	N-ter.	B, C	B) 54.5, C) 73.9
myotilin	**K 415**	actin	**D 27**	Ig2	A, B	A) 66.1, B) 57.6
myotilin	**K 303**	actin	**D 27***	Ig1	A	65.9
myotilin	**K 367**	actin	**D 365**	Ig2	B	63.6
myotilin	**K 367**	actin	**E 366**	Ig2	B	60.2
myotilin	**K 452**	actin	**E 363**	C-ter.	C	48.2
myotilin	**K 411**	actin	**D 26**	Ig2	A	47.8
myotilin	**K 474**	actin	**D 26**	C-ter.	C	35.4

*, cross-links shown on Fig 3; N-ter., region flanking Ig1Ig2 N-terminally; C-ter., region flanking Ig1Ig2 C-terminally; Score, computed as -10 log10 (E-value) representing best identification for a crosslink pair. The higher values of the score indicate more reliable identifications.
