# Supplementary material for: Molecular basis of F-actin regulation and sarcomere assembly via myotilin
Source: PLoS Biol. 2021 Apr 12;19(4):e3001148. doi: 10.1371/journal.pbio.3001148 (PMC8062120; doi:10.1371/journal.pbio.3001148)
Supplement: S3 Table — (PDF) [file pbio.3001148.s009.pdf]

S3 Table. List of constructs

Name	(1)Protein	Residues	Mutations	Vector	(2)Tag	Expression in	Origin
**Trx-MYOT**	myotilin	1-498	-	pETM-20	N-Trx-His_6-TEV	*E. coli*	this study
**Trx-MYOT-NEECK**	myotilin	1-498	F96E, L97E, L101E	pETM-20	N-Trx-His_6-TEV	*E. coli*	this study
**Ig1Ig2$^{185\text{-}498}$**	myotilin	185-498	-	pETM-20	N-Trx-His_6-3C C-Strep	*E. coli*	this study
**(3)Ig1Ig2$^{185\text{-}498}$***	myotilin	185-498	-	pDB-HisGST	N-His_6-GST-TEV	*E. coli*	this study
**Ig1Ig2$^{220\text{-}452}$**	myotilin	220-452	-	pETM-14	N-His_6-3C	*E. coli*	this study
**Ig1Ig2$^{220\text{-}452\ R405K}$**	myotilin	220-452	R405K	pETM-14	N-His_6-3C	*E. coli*	this study
**(4)Ig1Ig2$^{250\text{-}498}$**	myotilin	250-498	-	pETM-14	N-His_6-3C	*E. coli*	this study
**Ig1Ig2$^{250\text{-}444}$**	myotilin	250-444	-	pETM-14	N-His_6-3C	*E. coli*	[22]
**Ig1Ig2^{K354A}**	myotilin	250-444	K354A	pETM-14	N-His_6-3C	*E. coli*	this study
**Ig1Ig2^{Q356A}**	myotilin	250-444	Q356A	pETM-14	N-His_6-3C	*E. coli*	this study
**Ig1Ig2^{K358A}**	myotilin	250-444	K358A	pETM-14	N-His_6-3C	*E. coli*	this study
**Ig1Ig2^{K359A}**	myotilin	250-444	K359A	pETM-14	N-His_6-3C	*E. coli*	this study
**Ig1Ig2^{K367A}**	myotilin	250-444	K367A	pETM-14	N-His_6-3C	*E. coli*	this study
**Ig1Ig2^{R405K}**	myotilin	250-444	R405K	pETM-14	N-His_6-3C	*E. coli*	this study
**Ig1Ig2^{K411A}**	myotilin	250-444	K411A	pETM-14	N-His_6-3C	*E. coli*	this study
**Ig1Ig2$^{K354/359A}$**	myotilin	250-444	K354A, K359A	pETM-14	N-His_6-3C	*E. coli*	this study
**Ig1Ig2$^{K354/358/359A}$**	myotilin	250-444	K354A, K358A, K359A	pETM-14	N-His_6-3C	*E. coli*	this study
**Ig1$^{250\text{-}344}$**	myotilin	250-344	-	pET-3d(+)	N-His_6-TEV	*E. coli*	this study
**Ig2$^{349\text{-}459}$**	myotilin	349-459	-	pET-3d(+)	N-His_6-TEV	*E. coli*	this study
**MYOT^{WT}**	myotilin	1-498	-	pEGFP-N1	N-EGFP	C2C12	this study
**MYOT^{K354A}**	myotilin	1-498	K354A	pEGFP-N1	N-EGFP	C2C12	this study
**MYOT^{K359A}**	myotilin	1-498	K359A	pEGFP-N1	N-EGFP	C2C12	this study
**MYOT$^{K354/359A}$**	myotilin	1-498	K354A, K359A	pEGFP-N1	N-EGFP	C2C12	this study
**MYOT$^{K354/358/359A}$**	myotilin	1-498	K354A, K358A, K359A	pEGFP-N1	N-EGFP	C2C12	this study
**ACTN2-WT**	α-actinin-2	1-894	-	pET-3d(+)	N-His_6-TEV	*E. coli*	[7]
**ACTN2-NEECK**	α-actinin-2	1-894	R268E, I269E, L273E	pET-3d(+)	N-His_6-TEV	*E. coli*	[7]
**ACTN2-EF14**	α-actinin-2	746-894	-	pETM-14	N-His_6-3C	*E. coli*	this study
**palladin Ig3**	palladin	1022-1126	-	pTBSG	N-His_6-TEV	*E. coli*	[46]
**Doc2b**	Doc2b	125-412	-	pGEX4T1	N-GST-Thr	*E. coli*	[47]
**DVD-actin**	actin-5C	1-376	D287A, V288A, D289A	pFastBacHT	N-His_6	*Sf9*	[30]
**tropomyosin**	tropomyosin					*E. coli*	[33]

(1), myotilin (human, Uniprot-ID Q9UBF9); α-actinin-2 (human, P35609), palladin (Mus musculus, Q9ET54), Doc2b (Rattus norvegicus, P70610), actin-5C (Drosophila melanogaster, P10987), tropomyosin (Tpm1.1st, Homo sapiens, NP_001018005.1)

(2), in many cases removed during purification; N, N-terminal fusion; Trx, Thioredoxin-tag; His_6, 6xHis-tag, TEV; TEV protease cleavage site; 3C, HRV-3C protease cleavage site; C, C-terminal fusion; Strep, Strep-tag II; Thr, thrombin cleavage site, GST, Glutathione s-transferase-tag; EGFP, Enhanced green fluorescent protein-tag

(3), Ig1Ig2$^{185\text{-}498}$* was used to produce Ig1Ig2$^{185\text{-}454}$, and GST

(4), Ig1Ig2$^{250\text{-}498}$ was used to produce Ig1Ig2$^{250\text{-}466}$
