# Supplementary material for: Molecular basis of F-actin regulation and sarcomere assembly via myotilin
Source: PLoS Biol. 2021 Apr 12;19(4):e3001148. doi: 10.1371/journal.pbio.3001148 (PMC8062120; doi:10.1371/journal.pbio.3001148)
Supplement: Attachment — Submitted filename: Response to Reviewers.docx [file pbio.3001148.s010.docx]

Response to Reviewers

Please find below point-by-point reply to Reviewers’ comments and suggestions. In the manuscript and in this document, we highlighted the changes in the text addressing Reviewers’ comments in blue, **Reviewer’s comments are bold yellow highlighted**. A number of smaller editing changes used to shorten the manuscript and improve its flow/wording, are not highlighted in blue in order not to distract the reader/review.

# Reviewer #1:

**The research outlined in this paper is a comprehensive analysis of myotilin structure and a genuine effort has been put forth to gain a better understanding of myotilin’s mechanism of interaction with F-actin and other sarcomere proteins involved in Z-disc assembly. It is well written for the most part and uses a wide array of biochemical and structural analysis tools. Yet, I have major concerns about the conclusions drawn about the dimer structure as well as the opposing roles with respect to a-actinin put forth in the model (Fig. 7).**

*The manuscript was re-written to thoroughly address comments of all Reviewers. In this respect, the section in the dimer structure was removed and will be used as a part of another stand-alone publication as suggested by Reviewer #3. Only the concentration-dependent dimerization as assessed by the analysis of molecular parameters derived from one-dimensional SAXS scattering curve (Rg, Dmax, Mw) was retained in this manuscript.*

**The “summary” statement could be more strongly worded to emphasize the major findings of this research as opposed to list of results.**

*Following the Reviewer’s comments, the summary was re-written trying to emphasize the major findings. Furthermore, we included a statement of the significance of our research, page 2, line 44:*

# Significance Statement

Sarcomeres are the primary structural and functional unit of striated muscles, conferring movement in all animals. The Z-disk is the boundary between adjacent sarcomeres, where actin filaments (F-actin) are anchored. Z-disc protein myotilin, is a scaffold protein, which provides structural integrity to the Z-disc by multiple interactions to its central components, including F-actin and α-actinin-2. Here we provide the structure of myotilin, revealing its structural plasticity in solution and the first integrative structural model of its complex with F-actin. We further show that myotilin displaces tropomyosin from F-actin, implying a novel role of myotilin in sarcomere biogenesis beyond being an interaction hub for Z-disk partners.

**Introduction does not provide explanation of the previous data regarding myotilin dimerization (pg 3, line 20-21), nor does it explain which fragment can crosslink/bundle F-actin (pg 3, line 24).**

*Following Reviewer’s suggestion, we added mode details on previously published data reporting which fragment can crosslink/bundle F-actin and included some additional information on myotilin dimerisation. More information on published data regarding myotilin dimerization will be included in the manuscript dealing with the structure of myotilin dimer.*

*The amended text now reads (page 3, lines 94):*

... The shortest fragment of myotilin that can bind F-actin includes the two Ig domains preceded by a stretch of intrinsically disordered residues (aa 214-442) {von Nandelstadh, 2005 #698}. A longer myotilin fragment including N- and C-terminal regions flanking two Ig domains (aa 185-498) can cross-link actin filaments *in vitro* and *in vivo* {von Nandelstadh, 2005 #698}. In addition, full-length protein prevents F-actin disassembly induced by latrunculin A {Salmikangas, 2003 #68}, revealing its role in stabilization and anchoring of thin filaments in the Z-discs {Salmikangas, 2003 #68}. Myotilin dimerization was proposed to be important for F-actin cross-linking (Salmikangas et al., 2003). The shortest myotilin fragment with the ability to dimerize includes the Ig2 domain and the C-terminal tail (aa 345-498) {von Nandelstadh, 2005 #698}.

**The last paragraph of the introduction has one run-on sentence that needs to be rewritten for clarity.**

*We thank the Reviewer for pointing this out, the last paragraph was rewritten completely, and now reads (page 4, line 111):*

Based on our results we propose a model in which myotilin simultaneously binds F-actin and α-actinin with the concomitant displacement of tropomyosin. This renders myotilin not only structural support for Z-disc architecture but also a co-organiser of Z-disc assembly. Furthermore, our structural model of full-length myotilin provides a platform for understanding the molecular basis of disease-causing mutations and their impact on the structure, ligand binding and/or regulation.

**Results**

**There is no explanation in the text (or methods) for how the “sequence meta-structure analysis and disorder tendency analysis” were carried out. Was this a specific program? Need to describe this procedure or at least clearly reference the software used.**

*We changed the text in order to give a short explanation of the method used and added the reference, page 4, line 119:*

The previously performed disorder tendency analysis of full-length myotilin revealed that the N-terminal region of myotilin displays characteristics of an intrinsically disordered protein (IDP) [Puz et al., 2017]. The meta-structure analysis, which reflects how likely a specific residue is to be located within a compact 3D structure, was carried out as described previously [Konrat, 2009], and indicated a tendency for local compactions in the N-terminal ID region (**Figure 1B**).

*We accordingly amended the caption of Figure 1B:*

Large meta-structure derived compactness values indicate residue positions typically buried in the interior of the 3D structure, whereas small values are found for residues exposed to the solvent. Dashed line depicts the average compactness value (about 300) of the well-folded protein. Significantly smaller values (200) are found for structurally flexible proteins [Konrat, 2009].

**Authors do not explain why the full length Trx-MYOT is a monomer (SEC-SAXS + RALLS), whereas the truncated tandem Ig domains form concentration-dependent dimers.**

*We thank the Reviewer for pointing out that this was not clear in the original text. The full-length Trx-MYOT sample displays low solubility and cannot be brought to the concentrations at which dimerisation of truncated constructs is observed. This is the reason why Trx-MYOT is a monomer in SEC-SAXS + RALLS experiments.*

*We now explain why the full length Trx-MYOT is a monomer at the beginning of the section “Myotilin forms concentration-dependent dimers”, line 5, page 154:*

To investigate molecular determinants of dimerization we used SAXS on a series of truncated constructs: Ig1Ig2^250-444^ (Ig1Ig2 tandem), Ig1Ig2^220-452^ and Ig1Ig2^250-498^, due to the inherently low solubility of Trx-MYOT.

*In addition, we included new text the end of the section, to reiterate and make the point clearer, page 6, line 186:*

Our results together with published data on myotilin dimerization collectively show that: (i) N- and C-terminal regions flanking the Ig1Ig2 domains contribute to dimer stabilisation in solution; (ii) the fully dimeric form is observed only at high protein concentrations (> 1.0 mM), suggesting a weak association constant, explaining why only monomers of Trx-MYOT were observed in our SEC-SAXS experiments, where only low concentrations of Trx-MYOT could be used due to its aforementioned low solubility; (iii) tail-to-tail dimerisation reconciles both SAXS analysis and proposed mode of incorporation in the Z-disc, ochestered by interactions with filamin C and α-actinin-2 [Salmikangas, 2003].

**While the concentration-dependent trend in the SAXS data and the 15N-T2 transverse relaxation times do support a dimer, the authors should also consider using sedimentation equilibrium AUC for stronger evidence to support their dimerization data as this technique doesn’t require one to “assume a spherical particle” for analysis and could eliminate doubts raised by comparing proteins of different size (MW) and potentially different shape.** *We thank the Reviewer for s series of valuable suggestions; as we decided to remove the structure of the dimer from this manuscript, we will account for these Reviewer’s suggestions in the manuscript on myotilin structure and will not reply in detail here.*

**The XL-MS data reported in Table S2 shows a significant number of intermolecular crosslinks between Ig1 and Ig2 that the authors seem to ignore when they combined the XL-MS, NMR and SAXS data to generate their structural model of the dimer.**

*We removed the XL-MS data on myotilin dimer from this manuscript and will thoroughly attend to Reviewer’s comments in the manuscript on the myotilin dimer structure. We suspect that some of the cross-links that cannot be reconciled with the current dimer model can be due conformational dynamics due to the flexible linker between Ig1-Ig2 [Puz et al., 2017], and potentially to extended crosslinking reactions, which we will address experimentally.*

**In fact, I cannot figure out why they eliminate the possibility of a parallel dimer by assuming that it would result in polymerization. Parallel dimers could orient in such a way as to prevent polymerization just as well as antiparallel dimers.**

*We prepared a supplementary Figure S1E, S1F to illustrate the potential dimerisation modes and calculated theoretical Rg, P(r) and Dmax values for these models, for the reader to better understand how we interpret the data, as it was obviously not clear enough from the original text. The Reviewer is right that the parallel dimers could orient in such a way as to prevent polymerization, meaning that the absence of observed polymerisation does not necessarily exclude the parallel dimer arrangement, we therefore removed this argument from the manuscript.*

*In summary, we excluded parallel and anti-parallel dimers (SI1E, 2nd, 3rd from the left) since this arrangement cannot lead to a notable increase of Dmax (Figure SI1F). Based only on SAXS-derived molecular parameters, we cannot distinguish between staggered dimers, especially when taking into the account the conformational plasticity due to the flexible linker connecting Ig1 and Ig2 domains, leading to a structural ensemble [Puz et al, 2017]. Taking into account published data on myotilin dimerisation and function [Salmikangas et al, 2003] and our analysis here, the tail-to-tail arrangement is more plausible.*

*The main text was amended accordingly and supplementary Figures S1E-F added. Page 5 line 165:*

The notable increase of the D_max_ value in a concentration dependent manner from 110 Å to 150 Å together with aforementioned features of the *P(r)* suggests an antiparallel staggered dimer formed either by interactions *via* Ig1 (head-to-head) or *via* Ig2 domains (tail-to-tail), or a staggered parallel dimer *via* Ig1-Ig2. The calculated structural parameters (D_max_ and *P(r)*) of the non-staggered parallel or antiparallel dimer are not in line with the experimentally derived values, thus making such architecture less likely (**Figures 1F, S1E and S1F**).

*Please see also the text at the end of the section page 6, line 186, cited above.*

*Figure SI1E-F:*

*Figure Caption for SI1E-F:*

(**E**) Schematic representation of Ig1Ig2^220-452^ depicting potential modes of dimerization. (**F**) Theoretical *P(r)* *vs.* *r* plots calculated for potential Ig1Ig2^220-452^ dimerization modes showed in (**E**). Of these, both parallel and antiparallel dimers using tandem Ig1Ig2 as dimerization interface (parallel^(via Ig1Ig2)^, antiparallel^(via Ig1Ig2)^) display marginal increase in the D_max_. Staggered dimers display notable increase in the D_max_ similar to experimentally observed (**Figure 1F and Table S1**). In comparison to experimental data, where Ig1Ig2^220-452^ adopts a conformational ensemble in solution (**Figure 1E**) [Puz et al., 2017], *P(r)* *vs.* *r* plots for different dimerization modes were calculated using one (static) conformation, consequently resulting in a longer D_max_ compared to experimentally derived. In order to compare various *P(r)* functions, *P(r)* was normalized to the peak height.

**I was quite disturbed by the hand-waving regarding residue R405, which they claim is not involved in the dimer interface despite having the largest CSP because it just might be interacting with the disordered flanking regions.**

*After re-measurement and revision of our NMR-data (which are not shown here as they will be used in the manuscript on the myotilin dimer structure) R405 was found not to have the largest CSP. To address its contribution to dimerization we tested the effect of R405K mutation on dimer formation by SAXS, which showed that it does not have an impact on dimerization, as seen from the same concentration dependent dimerisation behaviour as displayed by the wild-type. The text was amended accordingly, and Figure SI1G added, which shows the comparison of P(r) plots for wild-type and R405K mutant in function of protein concentration. Page 5, line 180:*

R405 is mutated in muscular dystrophy, and was suggested to be responsible for defective homo-dimerization of myotilin {Shalaby, 2009 #33}. We therefore assessed the dimerisation propensity of Ig1Ig2^220-452 R405K^ mutant using SAXS. Comparative analysis of SAXS data and derived molecular parameters (D_max_, R_g_, *P(r)*, M_W_) revealed that the conserved R405K mutation which preserves the positive charge does not impair dimer formation *in vitro,* (**Figures S1G and Table S1**), in contrast to previously reported defective homo-dimerisation observed by immunoprecipitation and yeast two hybrid screens {Shalaby, 2009 #33}.

*Figure SI1G:*

*Figure Caption for SI1G:*

(**G**) *P(r)* *vs.* *r* plot for the concentration series of Ig1Ig2^220-452^ and Ig1Ig2^220-452 R405K^ measured at the same experimental conditions (for details see **Table S1**). Inset, respective concentration series with the corresponding SAXS profiles. Note, both Ig1Ig2^220-452^ and Ig1Ig2^220-452 R405K^, showed comparable concentration dependent increase in D_max_ suggesting that *in vitro* R405K mutation does not impair myotilin homo-dimerization.

**Using different buffer conditions for the actin binding assays makes it more difficult to compare the affinities with other ABPs and even within their constructs.**

*Different conditions were used in order to keep proteins soluble to the extent they can be used in the assay. Between sets of tested constructs (short ones IIg1^250-344^, Ig2^349-459,^ Ig1Ig2^250-444^, and Ig1Ig2^R405K^) and (long ones Trx-MYOT, Ig1Ig2^185-498^, Ig1Ig2^185-454,^ Ig1Ig2^250-498^, Ig1Ig2^250-444^) conditions were the same, allowing to compare affinities within the groups. In addition, IIg1Ig2^250-444^ was tested in both conditions and displayed similar apparent affinities, which is clearly stated in the text. We, therefore, trust that the conclusions from the data we made are possible and correct.*

*The text was amended to emphasise that the Ig1Ig2^250-444^ was tested in both conditions and gave comparable affinities, page 6, line 199:*

In the initial experiments (using conditions B1, for details, see Materials and Methods) with single Ig domains (Ig1^250-344^ and Ig2^349-459^), and their tandem (Ig1Ig2^250-444^), neither Ig1, nor Ig2 alone displayed significant binding to F-actin, while Ig1Ig2^250-444^ bound to F-actin with *K*_d_ = 9.2 ± 1.0 µM (**Figures 2A and S2A**). When the same experimental conditions were used for Trx-MYOT, its self-pelleting was observed making interpretation of the data difficult. To reduce the fraction of self-pelleted Trx-MYOT, we optimized the conditions for the co-sedimentation assays using a differential scanning fluorimetry (DSF)-based pH-screen (**Figure S2C**). We found that a slightly acidic pH, i.e. an adjustment of the assay buffer from pH 7.4 (in conditions B1) to pH 6.8 (conditions B2, for details, see Materials and Methods) rendered Trx-MYOT more stable as well as more soluble. To assess the effect of pH (6.8), affinity of Ig1Ig2^250-444^ to F-actin was determined using the conditions B2, too. The observed apparent *K*_d_ (12.9 ± 1.5 µM) was in good agreement with the affinity measured in the B1 conditions (*K*_d_ = 9.2 ± 1.0 µM) (**Figures 2A and S2B**, left panel), suggesting that diverse conditions used do not have a major effect on the myotilin affinity to F-actin.

**Why didn’t the authors examine the effect of R405K mutant on actin binding?**

*We did examine the effect of R405K mutant on actin binding. In the original manuscript F-actin binding of the R405K mutant was reported in Figure 3A, B):*

*These results are now reported in Figure 2A, and Figure SI2A. We highlighted corresponding text in the amended manuscript to facilitate reading, page 7, line 234:*

The muscular dystrophy-causing mutant R405K (Ig1Ig2^R405K^) [Shalaby et al., 2009] was also tested but did not show any notable difference in F-actin affinity with respect to the wild-type (**Figures 2A and S2A**), which agrees with its lateral location with respect to the identified positively charged patch involved in F-actin binding. Furthermore, the mutation retains the positive charge at this position (**Figure 2B**).

| *Figure 2A* | *Figure S2A* |
| --- | --- |
|  |  |

**What about bundling assays with their constructs?**

*We did not perform bundling/cross-linking assays in vitro. We were namely wondering how relevant this is for the function of the myotilin in vivo, where expression of myotilin starts at later stages of myogenesis when F-actin is already cross-linked with a-actinin and/or other actin cross-linking proteins [Salmikangas et al., 2003], leading to spacing of actin filaments of about 20 nm [*[*Oda et al, 2020*](https://www.biorxiv.org/content/10.1101/2020.04.14.041897v1)*;*  [*Burgoyne et al, 2019*](https://dx.doi.org/10.1073/pnas.1902235116)*], which is much longer than what could the myotilin dimer span. Thus in sarcomere myotilin can most likely bind only to already performed F-actin structures rather than perform cross-linking itself.*

**It is also not clear why the authors chose to use the dilution method for depolymerization assays instead of the more widely accepted LatA assay.**

*We used depolymerization assay induced by dilution, as it is a well established method that has been employed in a number of published studies (https://doi.org/10.1073/pnas.1001517107, https://doi.org/10.1091/mbc.E19-06-0317, https://doi.org/10.1073/pnas.1612719113) and is also recommended in the standard protocols for “Measuring Protein Binding to F-actin by Co-sedimentation” (doi:10.3791/55613).*

*The LatA assay was used for the full-length protein by Salmikangas et al., 2003 showing the same effect.*

**I would like to see the HSQC for the titration of F-actin into myotilin as opposed to just showing the CSP. I imagine that these spectra are almost indistinguishable and wonder if the CSP reported are significant.**

*As the Reviewer correctly anticipated, the CSP-values for the titration are quite small. HSQC for the titration of F-actin into myotilin Ig1^250-344^ and Ig2^349-459^ is now shown on Figure S3D.*

*In the analysis we refrained from interpreting single shifts but evaluated the pattern. Limits on expression yields and solubility as well as fast relaxation precluded us from measuring gradients with more F-actin for the full-length construct. The Ig-domain 1, however, which has more suitable exchange rates, was measured with larger amounts of F-actin which demonstrates that the shifts detected with low quantities of F-actin are indicative of stronger shifts observed with more F-actin. In addition, the most clearly distinguishable shifts match with the observed region in Ig1Ig2^250-444^.*

*The statistical significance of CSP-values was estimated by calculating residue-specific z-scores using the average chemical shift changes and the corresponding standard deviation. The z-score vs residue plot provided convincing evidence that the selected residues stretches are indeed most affected by the binding event and thus most likely part of the binding site. In all 4 regions residues with z-scores above 2 were detected, while no such large shift deviations were detected outside of the marked regions in Figure S3E, F.*

*Finally, we would like to point out that we used the available NMR-data only to provide additional indications regarding the validity of the model, rather than using it for model construction.*

*The main text was amended accordingly, page 8, line 287:*

Construct Ig1Ig2^250-444^ also showed a similar peak pattern, allowing the use of assignments of the single domains to obtain a partial assignment of this tandem construct. In the initial HSQC experiments, ^15^N-labelled Ig1^250-344^ was titrated with F-actin, which induced a reduction of the signal intensities (**Figure S3D**). Measurements of the ^15^N-labelled Ig2 domain (Ig2^349-459^) showed a stronger reduction in signal intensity upon addition of F-actin compared to those carried out with the Ig1 domain (**Figure S3D**) indicating stronger binding of Ig2 to F-actin.

*Figure S3D,E, F:*

*Caption to Figure S3D,E, F:*

(**D**) Relative change of ^1^H-^15^N HSQC cross-peak intensities upon addition of F-actin to Ig1^250-344^ (blue, left panel) or Ig2^349-459^ (red, right panel). Values were averaged by a sliding window function over 11 amino acids. Stronger reduction in signal intensity of Ig2^349-459^ as compared to Ig1^250-344^ indicates that Ig2 binds more tightly to F-actin than Ig1. In all experiments, except for the one indicated by an asterisk (67 μM), a fixed concentration of myotilin was used (100 μM), while the concentration of F-actin was varied as indicated in the figure. (**E**) ^1^H-^15^N HSQC monitored shift changes in Ig1Ig2^250-444^ (50 µM) upon addition of F-actin (4 µM). Shifts were averaged by a sliding window function over 11 amino acids. In Ig1Ig2^250-444^ four main regions with the most pronounced effect upon addition of F-actin were identified (boxed green). Inset, topology diagram of I-type Ig domain. (**F**) Shifts used in (**E**) expressed using z-scores (see Methods for pertinent details on calculation of z-score). For all regions indicated in (**E**) we find more than one shift with a z-score above one and at least one higher than two.

*Materials and Methods, NMR spectroscopy section now includes description of the calculation of the z-score, page 19, line 742:*

The interaction of Ig1^250-344^, Ig2^349-459^, Ig1Ig2^250-444^ with F-actin was monitored using ^1^H-^15^N HSQC measurements {Kay, 1992 #74;Palmer, 1991 #71}. Various concentrations were used in these experiments and are indicated in the corresponding figure legends (**Figure S3D and S3E**). Both shift perturbations and changes in peak intensity due to the addition of F-actin were followed. While intensity changes were monitored as a simple ratio between the signal intensities (I_bound_/I_free_), shifts were tracked as a weighted average between proton and nitrogen shift changes in peak centres. All figures show the root of the sum of squared proton and nitrogen shifts in ppm values. While the proton shift is taken “as is”, the nitrogen shift is divided by a factor of five in order to reflect the fact that dispersion in the nitrogen dimension is higher, while the achieved resolution is lower. Due to these circumstances, a diagonal shift has about 5 times higher changes in the nitrogen versus the proton dimension $shift=\sqrt{{\Delta H}^{2}+{(\frac{\Delta N}{5})}^{2}}$. In globular domains composed of β-sheets like Ig-domains of myotilin, chemical shift changes are typically less localised and more dispersed on residues through the hydrogen-bonding network stabilising adjacent β-strands in the β-sheet. We thus did not focus on individual residues, but rather on segments of residues, which are part of the interaction interface. Shift averages showing shift-patterns were therefore calculated by application of a rolling window average over 11 residues where a minimum of 2 values were required to obtain a value for the average. The z-scores given express the difference of the observed value (x) from the mean (µ) in terms of multiples of the standard deviation (σ), i.e. $z=\frac{x-\mu}{\sigma}$. The average shift µ was calculated as a simple average $\frac{\sum_{i=1}^{n} x}{n}$. The standard deviation σ was calculated as $\sigma=\sqrt{\frac{\sum_{i=1}^{n} {(x-\mu)}^{2}}{n-1}}$, n being the number of all observed values.

**It is somewhat suspect that “further experiments with 15N-labelled Ig1Ig2250-444 show a more prominent binding pattern upon addition of F-actin” as I am not sure what the data manipulation of “averaging shifts and scaling of identified regions” means.**

*Regrettably, we apparently did not describe our data treatment well. We present the data now with a running average of available shifts in an 11 amino acid window. The approach is now described extensively in M&M, page 20, line 755, please see the text above. The “further experiments” refer to the gradient with F-actin for the Ig1 domain and a similar shift pattern for measurements with F-actin and Ig2 separately, and are now presented in Figure S3D, please see the text above.*

**What “available data in the literature” was used to guide the docking (pg 10, line 7)? Please provide reference and describe briefly.**

*We thank the Reviewer for pointing this out. In effect, we did not use the literature data for docking, the modelling procedure is now described more in detail in the text and Materials and Methods section.*

*The integrative model is in agreement with and explains available published data, which showed that flanking regions contribute to binding as well [von Nandelstadh et al., 2005, Salmikangas et al., 2003]. We refer to this at the end of the paragraph.*

*Amended main text now reads, page 8, line 277:*

To construct an integrative molecular model of the myotilin:F-actin complex, we combined our structural model of Trx-MYOT (**Figure 1E**) and experimental data derived from *in vitro* mutational analysis and XL-MS, which we used as experimental distance restraints that guided the macromolecular docking using Haddock 2.2 [van Zundert et al., 2016] (for details see Material and Methods). In this model the tandem Ig domains land on the subdomains 1 of the adjacent actin subunits, with the flexible N- and C-terminal regions extending to the binding sites on neighbouring actin subunits as suggested by XL-MS data (**Figures 3B**), supporting and explaining published data [von Nandelstadh et al., 2005; Salmikangas et al., 2003].

*Materials and Methods, Macromolecular modelling section provides a detailed description of the modelling procedure, page 20, line 777:*

For the model of myotilin:F-actin complex the model of tandem Ig1Ig2 domains in semi-extended conformation based on SAXS data was used as a starting unbound structure [Puz et al., 2017]. Linker residues connecting myotilin Ig1 and Ig2 domains (residues 341-347), and N-terminal tail of actin (residues 3-6) were designated as flexible. As a starting F-actin structure an actin dimer was used (PDB 3J8I). Initial model was prepared by solvated docking implemented in Haddock 2.2 [van Zundert et al., 2016] using residues identified by chemical cross-linking and co-sedimentation assays as restraints. The best model with regard to Z-score was further refined using RosettaDock 2.3 [Lyskov et al., 2008] to yield the final myotilin:F-actin model.

**The peptide used in the study referenced (pg 12, line 10-11) was a palladin (not myotilin) sequence.**

*The peptide we refer to was indeed myotilin sequence as inferred from the text in [Beck et al., 2011], which reads (page 720, end of section ‘A general ligand recognition motif for α-actinin)’:*

*‘Further support for this binding motif is provided by NMR-based titration results (data not shown) with the myotilin and myopalladin peptide sequences, also containing this 1-4-5-8 motif, that result in CSPs of Act-EF34 that resemble those of palladin and titin.’*

**Discussion**

**Is the weak dimer biologically relevant? Would dimer of myotilin bind to F-actin, G-actin in vivo?** **Can you make any mutations based on model to disrupt the dimer and test this?**

*In order to address this question properly, in vivo experiments with mutants that cannot dimerize would be necessary, as the Reviewers suggest. We plan to address this question in the manuscript on myotilin dimer structure, which was removed from this manuscript.*

*Anyhow, regarding the weak dimerisation of myotilin we discuss molecular mechanisms of dimer formation in vivo, page 13, line 463:*

Our studies of myotilin dimerization in solution revealed that the regions flanking Ig1Ig2 domains enhance dimer stability. In comparison to the previous studies addressing myotilin dimerization [Salmikangas et al., 1999; Salmikangas et al., 2007; von Nandelstadh et al., 2005], we show that myotilin dimerizes in a concentration-dependent manner and that only concentrations higher than 1.0 mM showed complete dimer population, implying a weak association. This suggests mechanisms driving or stabilizing dimer formation *in vivo*: high local concentrations and avidity effects mediated by concominat binding to interaction partners such as α-actinin, filamin C and of course F-actin that teether myotilin to the Z-disc, and/or PTMs.

**Have the authors ever tried using Ig1 and Ig2, purified separately, but added to assays (binding, bundling, polymerization, depolymerization), to determine if linker or tandem domain structure is necessary for interactions with actin?**

*We did purify Ig1 and Ig2 separately as well as the tandem Ig domains and used these constructs in the F-actin binding assays (Figure 2A and Figure S2A, please see above). From these experiments, we can say that the tandem domain structure is necessary for the binding, but cannot conclude whether the linker contributes to the binding. Inspection of our XL-MS data does not reveal any cross-links between actin and the linker sequence.*

**Author contributions**

**Who did the experiments?**

*Author contributions (who did what), were added to the Author contribution section, following the guidelines* [*CRediT-taxonomy*](https://www.cell.com/pb/assets/raw/shared/guidelines/CRediT-taxonomy.pdf) *definitions.*

Conceptualization: KD-C, JK, BL and DOF; investigation: JK, MP, VP, TCS, FD, SM, MAG, CS, SS; methodology: JK, MP, VP, TCS, FD, SM, MAG, CS, SS, PFMV; visualization: JK, MP, TCS, MAG, PFMV; data curation: KD-C; supervision: KD-C, DOF, BW, RK, DIS, BL; validation: KD-C, DOF, BW, RK, PFMV, JK, DIS; resources: KD-C, DOF, BW, RK, DIS; funding acquisition: KD-C, DOF, BW, RK, BL; writing (original draft): JK, MP, VP, TCS, FD, SM, MAG, SS, PFMV, DOF, KD-C; writing (review and editing): all authors.

**Methods**

**It seems to me that the “manual selection” of the “best Ig2 dimer model” (pg 24, line 31) is quite biased. In fact, the use of only the Ig2 domain for the first stage of modeling is also biased. Why not start with docking of the tandem Ig1-Ig2 domain as both are known to be required?**

*The modeling of the dimer structure was apparently not clearly explained in the Materials and Methods section. In brief, we indeed started with docking Ig1-Ig2, Ig2 being the major dimerisation site as inferred from NMR experiments. In the next step of building Ig1-Ig2 dimer, we started from Haddock generated poses, and used complementary experimental information coming from SAXS, XL-MS and NMR to select the structural ensemble that best fit the data.*

*As mentioned before, this procedure will be explained in detail in the manuscript on myotilin dimer structure.*

**Figures**

**In Figure 1B, how was “compactness” determined? There is no explanation in the text (or methods) for how the “sequence meta-structure analysis and disorder tendency analysis” were carried out. Was this a specific program? Need to describe this procedure or at least clearly reference the software used.**

*Please see our answer to this Reviewer’s question above where we provide the description and report the text in the manuscript where the meta-structure analysis is explained.*

**Figure 7 - I can’t wrap my head around how myotilin can fulfill seemingly opposing roles with respect to a-actinin – in both stabilizing its interaction with F-actin, on one hand, and displacing it from F-actin, on the other hand.**

*We thank the Reviewer that Figure 7 is not clear. We therefore simplified Figure 7, which is now Figure 6, and focus on the tropomyosin displacement by myotilin.*

*Figure 6:*

**Figure S2A, please consider using different colors as the shades of purple are so similar that it is hard to detect two different sets of peaks in overlay.**

**Figure S2D, this figure is too small to be convincing in terms of showing where crosslinks were observed. Need to zoom in on critical areas.**

*We thank the Reviewer for this remark. We removed these figures from the manuscript, as they regarded experiments on dimerization. We followed the Reviewer’s advice and changed the colours in Figure S2A, and increased the size of the Figure S2D to improve its legibility and will use them in the study on the dimer structure.*

# Reviewer #2:

Olli Carpen, signed review

**This is a rather comprehensive manuscript on the structural aspects of myotilin and its interaction partners. Myotilin is one of the structural molecules of the sarcomere Z-disc, and missense mutations in myotilin cause muscle disorders. Myotilin has several binding partners, of which F-actin and a-actinin-2 are included in this study.**

**The study bases on the author's detailed knowledge of previous literature and well-defined research questions. The authors have set up to improve our understanding on the organisation of myotilin dimers and the consequences of dimerisation. They further show models of F-actin-myotilin interaction based on their experimental evidence, and how myotilin competes with tropomyosin for F-actin binding. Finally, they present a structural model of myotilin's organisation within the Z-disc.**

**The work is based on extensive experimental data with multiple complementary techniques. The manuscript is very clearly written, and main conclusions are understandable even for readers lacking deeper understanding of structural biology techniques.**

**I have very little criticise.**

**Perhaps one of the weaker points is the comparison between myotilin and palladin and their binding to PI(4,5)P2. Here the authors compare a single Ig-domain of palladin (which altogether has 5 Ig-domains) to a myotilin construct, which includes both its Ig-domains. It is difficult to rule out that the differences in PI(4,5)P2 binding would not be affected by the construct design.**

*Reviewer is right as according to published data “two palladin domains [immunoglobulin (Ig) 3 and 4] interact with the head group of PI(4,5)P2 with moderate affinity (apparent Kd = 17 μM).” [Yadav et al., 2016].*

*However, we used Ig3 palladin only as a positive control for PI(4,5)P2 binding, which served the purpose, while myotilin Ig1Ig2 did not interact with PI(4,5)P2 liposomes at the same experimental conditions.*

**Overall, I feel that this is a comprehensive structural manuscript on myotilin and its binding partners, which brings novel information on Z-disc biology, and which deserves to be published.**

# Reviewer #3:

**The manuscript “Structural insights into F-actin regulation and sarcomere assembly via myotilin” by Kostan et al presents a study of molecular interactions of myotilin, an important Z-disc structural protein associated with thin filaments in cardiac and skeletal muscles. Myotilin plays a crucial role in the organization and maintenance of the Z-disk assembly, and as such it is linked to myofibrillar myopathies, which lead to severe disabilities and death. Establishing myotilin functions is therefore essential for understanding of both healthy and diseased muscle structural organization.**

**The authors took a rather extensive experimental approach to dissect myotilin interactions in the Z-disc. The study was performed in several labs with complementary expertise, e.g. SAXS, NMR, CD, MS, FRAP, molecular modeling, biochemical assays, etc. A large swath of data was accumulated and presented in the paper in support of assertions made by the authors. Unfortunately, interpretations and conclusions are ridden with multiple problems. It is virtually impossible for a single Reviewer to have an in-depth understanding of and hand-on experiences with all the methodologies presented in the paper. Nevertheless, the imprecise manner of interpreting results and excessively brief description of the experimental details in the parts that I can comment on does not inspire confidence that everything else is done thoroughly, can be reproduced by other scientists, or has the meaning stated by the authors.**

**Specific examples**

**Regarding the statement that “Myotilin forms concentration-dependent antiparallel dimers”**

**Pps 5-6. “the larger construct showed an average twofold relaxation rate increase for residues in the Ig domains (Figure S2B). Assuming a spherical particle, a two-fold increase in 15N-R2 would indicate the diffusion rate of a two-fold more massive particle.” The second statement would be true if the dimer was the only species present in solution. However, according to another statement on p7 “(ii) the fully dimeric form is observed only at high protein concentrations (>1.8 mM), suggesting a weak association constant”. At the same time NMR experiments were performed at comparatively low protein concentrations (~50-200 uM, p23), which means that the fraction of a monomer in solution is considerable, and very likely to be dominant. Then the increase in transverse relaxation is a consequence of chemical exchange between monomeric and associated species, rather than simply a consequence of slower tumbling of single dimeric species. If that exchange is intermediate on the NMR chemical shift timescale, the chemical shifts would not change by much and would simply correspond to the monomeric chemical shifts, as was observed. In this scenario we cannot say from NMR data if it is a dimer that is forming or an oligomer or even a non-specific reversible/soluble aggregate (or all of the above). We also cannot say if the interactions are “electrostatically driven” and that chemical shift changes upon association are “minor”, because we just do not know the chemical shifts of associated species if their concentration is lower than the concentration of monomeric species in the sample. Additionally, if the associated species are polymers or aggregates, they would not be seen in the spectrum due to their size.**

*As already mentioned in response to Reviewer #1, the section in the dimer structure was removed and will be used as a part of another stand-alone publication as suggested by Reviewer #3. Only the concentration-dependent dimerization as assessed by the analysis of molecular parameters derived from one-dimensional SAXS scattering curve (Rg, Dmax, Mw) was retained in this manuscript.*

**The statement “a parallel dimer would result in a polymerization of myotilin molecules” on p6 is technically purely speculative, however the presented NMR data cannot refute such polymerization.**

*We agree that the NMR data cannot refute the polymerization, but our SAXS data (the method being very sensitive to presence of even small amounts of higher molecular weight species) do not show any higher oligomers formation in a concentration dependent manner.*

*We prepared a supplementary Figure S1E, S1F showing the potential dimerisation modes and calculated theoretical Rg, P(r) and Dmax values for these models, for the better understanding how we interpret the data, as it was obviously not clear enough in the original text. The Reviewer is right that the parallel dimers could orient in such a way as to prevent polymerization, meaning that the absence of observed polymerisation does not necessarily exclude the parallel dimer arrangement, we therefore removed this argument from the manuscript.*

*We excluded parallel and anti-parallel dimers (SI1E, 2nd, 3rd from the left) since this arrangement cannot lead to a notable increase of Dmax (Figure SI1F). Based only on SAXS-derived molecular parameters, we cannot distinguish between staggered dimers, especially when taking into the account the conformational plasticity due to the flexible linker connecting Ig1 and Ig2 domains, leading to a structural ensemble [Puz et al, 2017]. Taking into account published data on myotilin dimerisation and function [Salmikangas et al, 2003] and our analysis here, the tail-to-tail arrangement is more plausible.*

*The main text was amended accordingly and supplementary Figures S1E-F added. Page 5, line 165:*

The notable increase of the D_max_ value in a concentration dependent manner from 110 Å to 150 Å together with aforementioned features of the *P(r)* suggests an antiparallel staggered dimer formed either by interactions *via* Ig1 (head-to-head) or *via* Ig2 domains (tail-to-tail), or a staggered parallel dimer *via* Ig1-Ig2. The calculated structural parameters (D_max_ and *P(r)*) of the non-staggered parallel or antiparallel dimer are not in line with the experimentally derived values, thus making such architecture less likely (**Figures 1F, S1E and S1F**).

*Page 6, line 186:*

Our results together with published data on myotilin dimerization collectively show that: (i) N- and C-terminal regions flanking the Ig1Ig2 domains contribute to dimer stabilisation in solution; (ii) the fully dimeric form is observed only at high protein concentrations (> 1.0 mM), suggesting a weak association constant, explaining why only monomers of Trx-MYOT were observed in our SEC-SAXS experiments, where only low concentrations of Trx-MYOT could be used due to its aforementioned low solubility; (iii) tail-to-tail dimerisation reconciles both SAXS analysis and proposed mode of incorporation in the Z-disc, ochestered by interactions with filamin C and α-actinin-2 [Salmikangas et al, 2003].

*Figure SI1E-F:*

*Figure Caption for SI1E-F:*

(**E**) Schematic representation of Ig1Ig2^220-452^ depicting potential modes of dimerization. (**F**) Theoretical *P(r)* *vs.* *r* plots calculated for potential Ig1Ig2^220-452^ dimerization modes showed in (**E**). Of these, both parallel and antiparallel dimers using tandem Ig1Ig2 as dimerization interface (parallel^(via Ig1Ig2)^, antiparallel^(via Ig1Ig2)^) display marginal increase in the D_max_. Staggered dimers display notable increase in the D_max_ similar to experimentally observed (**Figure 1F and Table S1**). In comparison to experimental data, where Ig1Ig2^220-452^ adopts a conformational ensemble in solution (**Figure 1E**) {Puz, 2017 #6}, *P(r)* *vs.* *r* plots for different dimerization modes were calculated using one (static) conformation, consequently resulting in a longer D_max_ compared to experimentally derived. In order to compare various *P(r)* functions, *P(r)* was normalized to the peak height.

**If we nevertheless assume that only a dimer is formed upon association, the chemical shift changes shown in Fig.2c are too small (=<0.01-0.02 ppm) to be a reliable indicator of Ig2-Ig2 specific interactions in the putative dimer. They could be a consequence of pH variability between samples or, quite possibly, of transient interactions of the C-terminal extension (residues 445-498) with the Ig2 domain. How did the authors assure that a slight change in pH or the C-terminal extension effects on the chemical shifts of the monomer were not a factor? If it is the influence of the C-terminal extension on the chemical shifts of the monomer that we are observing, it is only natural that Ig2 is more affected than Ig1. None of these possibilities are discussed.**

*In regard to the Reviewers comments on the small chemical shift changes observed, we are happy to report that a new measurement of construct Ig1Ig2^250-498^ at higher concentration displayed higher chemical shifts than the data available at the point of submission. In the analysis of this data we now emphasize the shift pattern rather than individual shifts in order to avoid over-interpretation. As already mentioned, these data will be reported in the manuscript dealing with the structure of myotilin dimer.*

*In regard to a possible variability in pH we would like to note that the shifts are more prevalent in Ig2 than Ig1, and changes in pH would be expected to lead to shift changes distributed throughout the protein. The Reviewer correctly points out that the possibility of the C-terminal extension causing the observed changes in shift can’t be excluded from the shift data. However, SAXS, XL-MS and previous publications do point to a partial dimer in our sample that requires Ig2 for dimerisation [Salmikangas et al., 2003; von Nandelstadh et al.,, 2005], which is in line with the NMR-results.*

*The Reviewer is, however, correct that we should have made this assumption more clear and we will integrate the Reviewer's suggestion in the manuscript on myotilin dimerisation.*

**The picture of the 15N-HSQC spectra shown in S2A is of poor quality, but I can see a number of small peaks that have an appearance of impurities (e.g. weaker peaks in the 1H/15N~8.0/127.0 ppm area). Are all amide cross-peaks in the 250-498 fragment accounted for? If that’s an impurity I see, and it was copurified with the protein, can it cause the chemical exchange effects observed by the authors? If these peaks are not an impurity, but broadened peaks of the C-terminal extension (hard to tell given the figure quality), how is this consistent with the statement on p.5 “The additional C-terminal peaks of Ig1Ig2(250‑498) showed very high intensities compared to those of the Ig domains, indicating their unfolded character (Figure S2A)”? How is this statement consistent with the authors’ assertion that “the C-terminal extension is involved in dimerization” (p5, line 25)? Not clear nor adequately discussed.**

*We are happy to report that a new 15N-HSQC spectrum of the same construct (Ig1Ig2^250-498^) was re-measured after an improved expression and purification of the protein. The Reviewer was correct in pointing out that many weaker observed peaks are unlikely to be caused by the C-terminal extension. The corresponding peaks did stem from degradation of the protein and are not present in the new measurement. They were, however, not used for any data interpretation, and processing the data obtained with the new sample did not change our conclusions, but gave even better observables of the same changes.*

*We thank the Reviewer for pointing this out, and trust that the picture, which will be provided in the manuscript on myotilin dimerisation is of better quality. In the new measurement we still have a set of new peaks that are not assigned, as the assignment was transferred from the shorter Ig1 and Ig2 domains only. The changes observed between constructs Ig1Ig2^250-444^ and Ig1Ig2^250-498^, are now nearly exclusively adding high intensity peaks as is to be expected for a disordered C-terminal region.*

**To summarize, none of the presented NMR results are conclusive with respect to whether a dimer is formed and if it is, then if it is antiparallel. It appears that the results are explained in a way that fits the preferred narrative, while other possible explanations are ignored. Also, some statements contradict each other, which is probably a result of respective parts being written by different people and then mechanically combined. Can the conclusions of this section stand if NMR data are disregarded?**

*Indeed, the NMR data do not directly yield information on the parallel or antiparallel nature of interaction, which we actually drew from SAXS and XL-MS. In the revised manuscript we do not employ NMR data nor report the dimer structure but only describe the concentration-dependent dimerisation as assessed by the analysis of molecular parameters derived from one-dimensional SAXS scattering curve (Rg, Dmax, Mw).*

**“NMR mapping of myotilin residues binding to F-actin”**

**It is not clear to me why chemical shift perturbations are chosen to identify binding regions. The CSP (Fig. 4A) are extremely small (<~0.01 ppm) and could be a consequence of a small pH change upon F-actin addition. How were the samples prepared to ensure that there are no detectable effects due to pH variability? Following differential line intensity attenuation as a function of F-actin concentration would be more informative and convincing. The authors seem to have followed the peak intensity change (at a single actin concentration, as it appears) but never showed nor discussed their findings in detail.**

*It is true that the observed chemical shift perturbations are very small in our measurements. Unfortunately we are limited by the ability to achieve high concentrations of the longer constructs of myotilin possessing flanking regions and displaying higher affinity, in order to allow stronger changes to be observed in NMR experiments. As the changes are not distributed randomly throughout the protein it is very unlikely to be caused by pH changes, especially since both proteins (myotilin and F-actin) were dialysed into the same buffer prior to measurement.*

*As the Reviewer correctly points out that differential line intensity changes are often a valuable tool for investigating interactions, we do now report these changes for the interaction of myotilin Ig-domains with F-actin in the supplementary information. HSQC for the titration of F-actin into myotilin Ig1^250-344^ and Ig2^349-459^ is now shown on Figure S3D. As we already stated in reply to Reviewer #1:*

*In the analysis we refrained from interpreting single shifts but evaluated the pattern. Limits on expression yields and solubility as well as fast relaxation precluded us from measuring gradients with more F-actin for the full-length construct. The Ig-domain 1, however, which has more suitable exchange rates, was measured with larger amounts of F-actin which demonstrates that the shifts detected with low quantities of F-actin are indicative of stronger shifts observed with more F-actin. In addition, the most clearly distinguishable shifts match with the observed region in Ig1Ig2^250-444^.*

*The statistical significance of CSP-values was estimated by calculating residue-specific z-scores using the average chemical shift changes and the corresponding standard deviation.*

*The z-score vs residue plot provided convincing evidence that the selected residues stretches are indeed most affected by the binding event and thus most likely part of the binding site. In all 4 regions residues with z-scores above 2 were detected, while no such large shift deviations were detected outside of the marked regions in Figure 3E, F.*

*Finally we would like to point out that we used the available NMR-data only to provide additional indications regarding the validity of the model, rather than using it for model construction.*

*The main text was amended accordingly, page 8, line 284:*

NMR was subsequently used to validate the structural model of myotilin:F-actin complex. NMR spectra of the isolated ^15^N-labelled Ig1 and Ig2 domains showed similar characteristics to those available in the BMRB databank (Ig1: 7113; Ig2: 16370) [32], and hence *de novo* assignment was not required in order to obtain a subset of assigned peaks. Construct Ig1Ig2^250-444^ also showed a similar peak pattern, allowing the use of assignments of the single domains to obtain a partial assignment of this tandem construct. In the initial HSQC experiments, ^15^N-labelled Ig1^250-344^ was titrated with F-actin, which induced a reduction of the signal intensities (**Figure S3D**). Measurements of the ^15^N-labelled Ig2 domain (Ig2^349-459^) showed a stronger reduction in signal intensity upon addition of F-actin compared to those carried out with the Ig1 domain (**Figure S3D**) indicating stronger binding of Ig2 to F-actin.

Further experiments with ^15^N-labelled Ig1Ig2^250-444^ showed a shift pattern upon addition of F-actin, where the regions with significant HSQC shifts are: 257-270, 296-309, 363-374 and 405-413 (**Figure S3E and S3F**) with segment 301-306 unfortunately not being represented due to low signal intensity [32]. These regions map to the ‘BED’ β-sheet of both Ig domains and additionally to the β-strand A’ in Ig1, and are in agreement with the interaction sites mapped by binding, mutational and XL-MS data (**Figure 2A, 2D and 3A**).

*Figure S3D,E, F:*

*Caption to Figure S3D,E, F:*

(**D**) Relative change of ^1^H-^15^N HSQC cross-peak intensities upon addition of F-actin to Ig1^250-344^ (blue, left panel) or Ig2^349-459^ (red, right panel). Values were averaged by a sliding window function over 11 amino acids. Stronger reduction in signal intensity of Ig2^349-459^ as compared to Ig1^250-344^ indicates that Ig2 binds more tightly to F-actin than Ig1. In all experiments, except for the one indicated by an asterisk (67 μM), a fixed concentration of myotilin was used (100 μM), while the concentration of F-actin was varied as indicated in the figure. (**E**) ^1^H-^15^N HSQC monitored shift changes in Ig1Ig2^250-444^ (50 µM) upon addition of F-actin (4 µM). Shifts were averaged by a sliding window function over 11 amino acids. In Ig1Ig2^250-444^ four main regions with the most pronounced effect upon addition of F-actin were identified (boxed green). Inset, topology diagram of I-type Ig domain. (**F**) Shifts used in (**E**) expressed using z-scores (see Methods for pertinent details on calculation of z-score). For all regions indicated in (**E**) we find more than one shift with a z-score above one and at least one higher than two.

*Materials and Methods, NMR spectroscopy section now includes description of the calculation of the z-score, page 19, line 742:*

The interaction of Ig1^250-344^, Ig2^349-459^, Ig1Ig2^250-444^ with F-actin was monitored using ^1^H-^15^N HSQC measurements [Kay et al., 1992; Palmer et al., 1991]. Various concentrations were used in these experiments and are indicated in the corresponding figure legends (**Figure S3D and S3E**). Both shift perturbations and changes in peak intensity due to the addition of F-actin were followed. While intensity changes were monitored as a simple ratio between the signal intensities (I_bound_/I_free_), shifts were tracked as a weighted average between proton and nitrogen shift changes in peak centres. All figures show the root of the sum of squared proton and nitrogen shifts in ppm values. While the proton shift is taken “as is”, the nitrogen shift is divided by a factor of five in order to reflect the fact that dispersion in the nitrogen dimension is higher, while the achieved resolution is lower. Due to these circumstances, a diagonal shift has about 5 times higher changes in the nitrogen versus the proton dimension $shift=\sqrt{{\Delta H}^{2}+{(\frac{\Delta N}{5})}^{2}}$. In globular domains composed of β-sheets like Ig-domains of myotilin, chemical shift changes are typically less localised and more dispersed on residues through the hydrogen-bonding network stabilising adjacent β-strands in the β-sheet. We thus did not focus on individual residues, but rather on segments of residues, which are part of the interaction interface. Shift averages showing shift-patterns were therefore calculated by application of a rolling window average over 11 residues where a minimum of 2 values were required to obtain a value for the average. The z-scores given express the difference of the observed value (x) from the mean (µ) in terms of multiples of the standard deviation (σ), i.e. $z=\frac{x-\mu}{\sigma}$. The average shift µ was calculated as a simple average $\frac{\sum_{i=1}^{n} x}{n}$. The standard deviation σ was calculated as $\sigma=\sqrt{\frac{\sum_{i=1}^{n} {(x-\mu)}^{2}}{n-1}}$, n being the number of all observed values.

**On p24 the authors imply (although do not explicitly say) that Ig1-Ig2(250-444) interactions with actin are in fast exchange. With the Kd estimate of ~10 uM this might be not the case, and the observed chemical shifts (assuming that the effect of pH upon F-actin addition is negligible and the only cause of the shifts is interactions with actin) would not be the population-averaged values between the free protein and the complex. Does it affect any conclusions regarding “restraints obtained from NMR”? Not adequately discussed.**

*We here wanted to emphasize our reasons to use Ig1-Ig2(250-444), rather than the longer construct Ig1-Ig2(250-498) despite the latter having higher affinity for F-actin. The Reviewer is right that a slow exchange would prohibit us from seeing effects of F-actin binding on myotilin. A Kd of about 10 µM is, however, not prohibitive of fast exchange being present in the system under investigation and a value of about 3 µM is seen as a “rule of thumb” for fast exchange, which can be higher or lower depending on K_on_, K_off_, field strength, observed nucleus and the shift difference of the free and bound forms (*[*https://doi.org/10.1016/j.pnmrs.2013.02.001*](https://doi.org/10.1016/j.pnmrs.2013.02.001)*). In addition we detect similar shift regions on the single Ig1 domain, which has a much lower affinity to F-actin and, therefore, is clearly in the fast exchange regime. Higher concentrations were necessary in order to obtain similar averaged shift values.*

*A figure below is added for the Reviewer:*

*The figure shows shifts with window size 11 of 50µM Myot 250-444 with 4µM F-actin and compares it to averaged shifts obtained from 86µM Myot Ig1 250-344 and 52 µM F-actin as these are of comparable magnitude. The values for 250-444 are as in Fig S3E while the shift values of 250-344 stem from the sample used for height comparisons in S3D.*

**“Integrative structural model of the myotilin:F-actin complex”**

**Information on how NMR was used to obtain distance restraints is limited to a brief statement in the Methods “Initial restraints were obtained from chemical cross-linking, NMR and co-sedimentation assays.” A standard way to obtain NMR distance restraints is via NOE. Short of that, a more detailed justification and explanations are warranted.**

*Regarding the “restraints obtained from NMR” we thank the Reviewer for catching this error, we have not used NMR-data to generate the model of F-actin/myotilin, but only used the NMR data to show its validity subsequently. This is the reason no NMR-restraints are described in the methods section. We have edited this error in the amended manuscript. The main text was amended accordingly, page 8, line 277:*

To construct an integrative molecular model of the myotilin:F-actin complex, we combined our structural model of Trx-MYOT (**Figure 1E**) and experimental data derived from *in vitro* mutational analysis and XL-MS, which we used as experimental distance restraints that guided the macromolecular docking using Haddock 2.2 [31] (for details see Material and Methods). In this model the tandem Ig domains land on the subdomains 1 of the adjacent actin subunits, with the flexible N- and C-terminal regions extending to the binding sites on neighbouring actin subunits as suggested by XL-MS data (**Figures 3B**), supporting and explaining published data [10, 17].

NMR was subsequently used to validate the structural model of myotilin:F-actin complex. NMR spectra of the isolated ^15^N-labelled Ig1 and Ig2 domains showed similar characteristics to those available in the BMRB databank (Ig1: 7113; Ig2: 16370) [32], and hence *de novo* assignment was not required in order to obtain a subset of assigned peaks.

*The the description of macromolecular modelling in Material and methods is corrected and provides more detail, the amended text, page 20, line 771 now reads:*

## Macromolecular modeling

The Ig1Ig2^220-452^ models displaying hypothetical dimer architectures were prepared by manually moving two identical subunit copies where the Ig1 and Ig2 domains were in an extended relative orientation. The two subunits were oriented in a way to mimic Ig-Ig interactions, which would support dimer formation and to minimize steric clashes. These models were then used to calculate scattering profile using Crysol [Svergun et al., 1995], and *P(r)* using GNOM [Svergun et al., 1992], both part the ATSAS software package [Franke et al., 2017].

For the model of myotilin:F-actin complex the model of tandem Ig1Ig2 domains in semi-extended conformation based on SAXS data was used as a starting unbound structure {Puz, 2017 #6}. Linker residues connecting myotilin Ig1 and Ig2 domains (residues 341-347), and N-terminal tail of actin (residues 3-6) were designated as flexible. As a starting F-actin structure an actin dimer was used (PDB 3J8I). The initial model was prepared by solvated docking implemented in Haddock 2.2 {van Zundert, 2016 #97} using residues identified by chemical cross-linking and co-sedimentation assays as restraints. The best model with regard to Z-score was further refined using RosettaDock 2.3 {Lyskov, 2008 #75} to yield the final myotilin:F-actin model.

**Figure S2B. R2 units of measurement are Hz (inverse seconds, not seconds), the axis label should read 15N R2/Hz**

*We thank the Reviewer for pointing out that the label of the axis was easily misunderstood. It was intended to read “R2/s” = “R2 per second” but we have changed it to read “[s^-1^]” in order to clearly indicate the units used. We are not using Hz as these are usually employed for recurring (cyclic) processes, which is not the case here. This figure was removed from this manuscript and will be used in the amended version in the manuscript on myotilin dimerisation.*

**The display of CD spectra of mutants (Fig. S3C) is too poor of quality to make a judgment if there were no structural changes caused by mutations. We can only conclude that the mutants did not completely unfold, which is trivial information given the fact that binding was not completely abolished. More subtle local changes are hard to see from these spectra, particularly given the fact that there is something obviously wrong with the R405K spectrum. The ellipticity is clearly negative at 240 nm, which should not have happened. Were reference buffer spectra properly subtracted? If not, would that make the conclusion on p.8 that “mutants did not show any [detectable] differences in secondary structure content…” inaccurate? If it were inaccurate, would the conclusion of purely electrostatic nature of interactions be wrong as well? How does that affect assumptions that went into generating distance restraints for the myotilin/F-actin complex?**

*We recognised that, as pointed out by the Reviewer, the quality of the CD data was not good enough to justify possible, subtle structural changes caused by mutations, and removed the CD data from this manuscript. Given that all the mutated residues map to the Ig-domain surface and are solvent-exposed, we think we have reasons to assume that the mutations will not affect the overall fold of the domain, as also pointed by the Reviewer. Indeed we did not notice any altered behaviour of mutants in size-exclusion chromatography, which is sensitive to the hydrodynamic radius of macromolecules, nor did the solubility of the constructs change.*

**The paper cannot be published in its current form. To some extent it is understandable that with this number of experiments and the manuscript being already almost 8000 words, a sufficiently detailed account of the performed experiments and proper discussion of the experimental results would make the size of the paper unmanageable. However, without these details, presented broad assertions of biological significance are not convincing. In my opinion, the MS should be broken in 2-3 parts, each with fewer messages. The authors should spend more effort on demonstrating to readers that their specific conclusions are correct, only then claims of biological significance can be adequately assessed.**

*We followed Reviewers’ suggestions and split manuscript into two parts (myotilin/F-actin interaction and structure of myotilin dimer); we put an effort in streamlining the text, manuscript readability and focused on a smaller number of messages with improved clarity.*
