# Supplementary material for: Molecular basis of F-actin regulation and sarcomere assembly via myotilin
Source: PLoS Biol. 2021 Apr 12;19(4):e3001148. doi: 10.1371/journal.pbio.3001148 (PMC8062120; doi:10.1371/journal.pbio.3001148)
Supplement: Attachment — Submitted filename: Reply_to_Reviewers.docx [file pbio.3001148.s012.docx]

Dear Senior Editor Ines Alvarez-Garcia, dear Reviewers

Please find below the reply to Reviewer #1 comments and suggestions. All changes are highlighted in blue through the text, both in the manuscript and in this document.

In order to alleviate the job of the Editor and Reviewers, we added links to 3D-models of the structures shown in Figures 1, 2, 4, 6, and S3 in the corresponding legends.

Best wishes

Kristina Djinovic Carugo

# Reviewer #1

# *We thank Reviewer #1 for her/his careful reading of our manuscript and the many insightful comments and suggestions, which we trust improved substantially the manuscript. We added a sentence of acknowledgment in the Acknowledgment section, appreciating Reviewer #1 invested time and comments.*

As a reviewer of the previous manuscript submitted by these authors entitled "Structural insights into F-actin regulation and sarcomere assembly via myotilin," I am pleased by the significantly modified manuscript now under review. The authors have made a good effort to modify the manuscript with the suggestions from all three previous reviewers, including the removal of most results and discussion regarding evidence of dimerization. They intend to publish this part as a separate story and the greatly simplifies the complexity of their current manuscript. Yet, they still include some discussion of the dimerization data and continue to advocate for the tail-to-tail dimerization model with minimal data to support this staggered arrangement over the parallel model. While the authors have provided more rationale and potential modes of dimerization in S1E, the experimentally determined Dmax values of ~152 (Table S1) at the highest concentration don't eliminate any of the "staggered" model predicted values (159-179).

*The reviewer is right, the molecular parameters derived from analysis of the experimental SAXS data, compared to the theoretically predicted values do not allow to distinguish between staggered models, but to exclude the non-staggered parallel and antiparallel dimer. We advocated for the tail-to-tail model as this structural model is in line with the model of incorporation of myotilin in the Z-disc, proposed by Salmikangas et al, 2003. Following Reviewer #1 comment, we toned down the tail-to-tail dimerization model, and modified the sentence at the of the section “Myotilin is a conformational ensemble in solution section”, line 189:*

Although tail-to-tail dimerization reconciles both SAXS analysis and proposed mode of incorporation in the Z-disc, orchestrated by interactions with filamin C and α-actinin-2 {Salmikangas, 2003 #17}, further higher resolution structural studies are needed to describe the quaternary structure of myotilin in detail.

Authors do admit that the CSP values for the titration of F-actin into myotilin are "quite small," and claim to include the HSQC in Figure S3D. However, this data is an abstraction or "interpretation of patterns" of the raw data and I am not sure this is an acceptable practice in the field.

*There seems to be some misunderstanding with the data shown in Figure S3. Figure S3D shows the reduction in signal intensities as a function of protein concentrations. Reduction of signal intensities (extracted from HSQC data) is due to protein interactions and the formation of particles with increased molecular weight (resulting in shorter transverse relaxation times and concomitant lower intensities). Since signals in Ig2 decrease significantly faster with increasing protein concentration this indicates that the affinity of the Ig2 domain is higher compared to Ig1.*

*With respect to CSP values: Figure S3E shows chemical shift perturbation profiles (CSP as a function of residue position) and reveals four distinct binding site regions (indicated by green boxes). Data presentation/analysis was performed as established in biomolecular NMR spectroscopy. However, given that the observed CSP were found to be rather small a more sophisticated statistical analysis (Figure S3F, z-score values) was performed to validate the findings and provide a statistical argument for the significance of the extracted binding site regions.*

*Please note that the old Figures S3D, E, F are in the amended manuscript Figures S3E, F, G.*

Figure 3 in the text has been improved, but it should be noted that very few data restraints from NMR or "co-sedimentation" (which should be mutagenesis) inform the structural model for binding to F-actin. So few, that I question whether Figure 3B really should be included as I don't think it adds much beyond Figure 3A and is repeated to some degree again in model in Figure 6.

*Following Reviewer #1 suggestion, we moved Figure 3B to the Supplementary Figures, and is now Figure S3D. We thank re reviewer for noting the mistake having "co-sedimentation" twice, which we replace with mutagenesis.*

Figure 6 legend also needs to be revised to better explain what the red and blue/purple balls are. There is also an unclosed parenthesis, too.

*Following Reviewer #1 suggestion, added information on regarding the red and blue/purple balls, and added the following sentence to the caption for the Figure 6:*

α-actinin‑2 dimers are schematically shown in an open conformation bound to myotilin, with ABD highlighted as red spheres, and EF12 and EF34 of CAMD as violet and purple spheres, respectively. Myotilin in tropomyosin are shown in colour code as used in **Figure 1 and 5**.

Most of the main claims of this paper are based on the structural model developed from several different structural studies, including SAXS, NMR, XL-MS and mutagenesis; however, I think that the authors overstate the claim that this model "provides a platform for understanding the molecular basis of disease-causing mutations…" First of all, most mutations lie in the N-terminal region which is not resolved in their model at all. The only exception is the R405K mutation which they show does not impair homodimerization or actin binding, leaving this mutation still a mystery as to effect on function.

*Following Reviewer #1 comment, we removed this statement "…provides a platform for understanding the molecular basis of disease-causing mutations…" from the text.*

I did approve of the changes to the summary statement and inclusion of a "statement of significance" which is accurate and highlights what I feel is the most interesting result regarding the displacement of tropomyosin by myotilin.

The data better described in the context of current literature and I feel that the manuscript will be of interest to scientists in this field like myself.

With the revisions to the text, I think this paper is much closer to being acceptable for publication. The main concerns that I still have revolve around the dimerization models and weak data to support some claims made regarding interpretation of the structural model in general.
